# Blood, sweat, and tears: extraterrestrial regolith biocomposites with *in vivo* binders

**DOI:** 10.1016/j.mtbio.2021.100136

**Published:** 2021-09-10

**Authors:** A.D. Roberts, D.R. Whittall, R. Breitling, E. Takano, J.J. Blaker, S. Hay, N.S. Scrutton

**Affiliations:** a Manchester Institute of Biotechnology and Department of Chemistry, The University of Manchester, M1 7DN, UK; b Department of Materials and Henry Royce Institute, The University of Manchester, Manchester M13 9PL, United Kingdom; c Department of Biomaterials, Institute of Clinical Dentistry, University of Oslo, Oslo 0317, Norway

**Keywords:** Human serum albumin, Hybrid materials, In situ resource utilization, Biopolymer-bound soil composites, Recombinant spider silk, 3D-printing, ERB, Extraterrestrial Regolith Biocomposite, HSA, Human Serum Albumin, BSA, Bovine Serum Albumin, MSA, Mammalian Serum Albumin, LHS-1, Lunar Highlands Simulant 1, MGS-1, Martian Global Simulant 1, CD, Circular Dichroism, FE-SEM, Field-emission Scanning Electron Microscopy

## Abstract

The proverbial phrase ‘you can’t get blood from a stone’ is used to describe a task that is practically impossible regardless of how much force or effort is exerted. This phrase is well-suited to humanity’s first crewed mission to Mars, which will likely be the most difficult and technologically challenging human endeavor ever undertaken. The high cost and significant time delay associated with delivering payloads to the Martian surface means that exploitation of resources *in situ* — including inorganic rock and dust (regolith), water deposits, and atmospheric gases — will be an important part of any crewed mission to the Red Planet. Yet there is one significant, but chronically overlooked, source of natural resources that will — *by definition* — also be available on any crewed mission to Mars: the crew themselves. In this work, we explore the use of human serum albumin (HSA) — a common protein obtained from blood plasma — as a binder for simulated Lunar and Martian regolith to produce so-called ‘extraterrestrial regolith biocomposites (ERBs).’ In essence, HSA produced by astronauts *in vivo* could be extracted on a semi-continuous basis and combined with Lunar or Martian regolith to ‘get stone from blood’, to rephrase the proverb. Employing a simple fabrication strategy, HSA-based ERBs were produced and displayed compressive strengths as high as 25.0 MPa. For comparison, standard concrete typically has a compressive strength ranging between 20 and 32 MPa. The incorporation of urea — which could be extracted from the urine, sweat, or tears of astronauts — could further increase the compressive strength by over 300% in some instances, with the best-performing formulation having an average compressive strength of 39.7 MPa. Furthermore, we demonstrate that HSA-ERBs have the potential to be 3D-printed, opening up an interesting potential avenue for extraterrestrial construction using human-derived feedstocks. The mechanism of adhesion was investigated and attributed to the dehydration-induced reorganization of the protein secondary structure into a densely hydrogen-bonded, supramolecular β-sheet network — analogous to the cohesion mechanism of spider silk. For comparison, synthetic spider silk and bovine serum albumin (BSA) were also investigated as regolith binders — which could also feasibly be produced on a Martian colony with future advancements in biomanufacturing technology.

## Highlights

• A protein from human blood can form a biocomposite material with the moon or Mars’ dust.
• The compressive strength of the biocomposite materials is on par with concrete.
• Incorporating urea (from urine) can increase the compressive strength by over 300%.
• We demonstrate that the resulting biocomposites can potentially be 3D-printed.

## Graphical abstract

**Figure:**
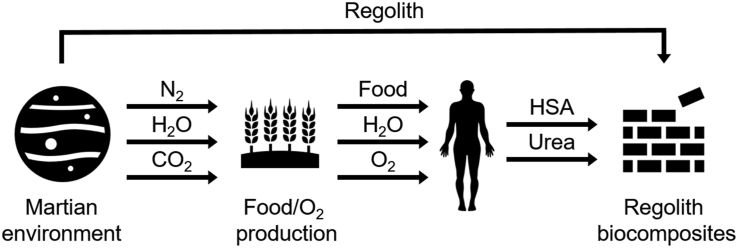

## 1 Introduction

Without an appreciable atmosphere or magnetosphere for protection, exposure to galactic cosmic radiation, solar particle events and micrometeor strikes poses a significant survival risk to any crewed mission to Mars or long-term mission to the Lunar surface [1,2]. Due to the high cost of extra-orbital payload delivery, early extraterrestrial colonies will likely exploit loose unconsolidated rock and dust (also known as regolith) as a bulk material for radiation and meteor shielding [2]. The loose, particulate nature of Lunar and Martian regolith will likely necessitate some form of mechanical stabilization to prevent erosion from high-velocity exhaust plumes or Martian dust storms — wind speeds on Mars can exceed 100 km per hour [3]. Strongly bonded extraterrestrial regolith, combined with a pressurized internal liner, could also serve as a cost-effective construction material for habitat expansion [4]. Several NASA-backed companies are currently developing additive manufacturing (3D-printing) technologies for extraterrestrial habitat construction based on *in situ* regolith with suitable binders [5].

Although regolith binders could be produced on Earth (*ex situ*) and delivered to the colonies, if binders could be produced *in situ* using resources available onsite (so-called *in situ* resource utilization, ISRU), this could provide a significant reduction in payload mass and mission cost [2]. The feasibility of producing *in situ* binders based on biological, synthetic polymer, inorganic, or cement-based materials has been explored and evaluated by others [[6], [7], [8]]. Numerous significant hurdles still remain before such approaches can realistically be employed — largely due to heavy equipment and spare parts needing to be transported from Earth, which would increase mission complexity and largely offset the benefit of employing ISRU [6]. For a crewed mission to Mars, any such equipment would also have to guarantee ultra-high reliability and redundancy since the delivery of any replacement equipment or components from Earth will be restricted by limited launch windows and long travel times [1]. The exploitation of natural resources produced by the astronauts themselves (*in vivo*) has received surprisingly little attention, despite the ready availability — *by definition* — of such resources on any crewed mission.

Adhesives and binders of biological origin were widely utilized by humanity for millennia before the development of synthetic petroleum-derived adhesives. Tree resins, collagen from hooves, casein from cheese, and animal blood were all used as binders and additives for various applications [[9], [10], [11]]. With relatively recent advances in our understanding of protein structure and function, the ability to create designer proteins through synthetic biology, and the need to move away from petrochemical-based feedstocks, there is a renewed interest in employing biopolymers for material applications and construction [[12], [13], [14], [15], [16], [17]].

In this work, we explore the use of human serum albumin (HSA) as a binder for the fabrication of ERBs. We found that ERBs comprised of nothing but simulated moon or Mars regolith, HSA, and water could have ultimate compressive strengths (UCSs) as high as 25.0 MPa — which compares exceptionally well against other proposed regolith stabilization technologies (Table 1). HSA is the most abundant protein in human blood plasma, present at a concentration of 40–45 g L^−1^, and is replenished at a rate of 12–25 g day^−1^ in healthy adults [18]. HSA could be obtained from crew members through blood plasma extraction — an established procedure on Earth, which can safely be performed multiple times a week via a procedure similar to blood donation [19]. If HSA-ERBs were utilized as a mortar and combined with a sandbag-based construction method, our calculations suggest that each crew member — over the course of a 72-week Mars mission — could produce enough HSA to construct habitat-space to support an additional astronaut (details of calculation in SI). This could allow the steady expansion of a nascent Martian colony. Unlike other proposed binder materials, HSA production does not require any additional synthesis technology such as bioreactors or synthetic polymer/resin production equipment — which would add additional mass (and therefore expense) to a Martian mission, as well as increase energy-, water-, and workload-demand, and also be susceptible to component failure. Aside from acting as a mortar-like binder for sandbag-based construction, we also demonstrate the potential for HSA-ERBs to be 3D-printed, which could be a more feasible construction method on Mars and was a target of a recent centennial competition hosted by NASA [5]. We also demonstrate that HSA can strongly adhere to glass, meaning that melted or sintered glassy regolith bricks could potentially be bonded together by HSA-based adhesives or HSA-ERB mortars.

**Table 1:** Comparison of the mechanical properties and processing energy requirement of several other regolith stabilization technologies, including primary disadvantages of each technique. Note that quantitative data for processing energy requirements were not available for all sources, so qualitative data (low/medium/high) were presented in these instances.

Method	UCS (MPa)	Processing energy (kWh/MT)	Primary disadvantages	Refs.
Melted and cast regolith	550	360 (very high)	Extremely high processing energy and temperature (1200–1500 °C)	[7,20]
Sintered regolith	14.5	156 (high)	High processing energy and temperature (1000–1200 °C)	[7,20,21]
Extraterrestrial concrete	75.5	High	High processing energy and water consumption. Geographically sparse precursors	[7]
Sulfur-bound regolith	30	High	High processing energy. Susceptible to sublimation	[7,20]
Sand-bagging	2	Low	Poor mechanical properties. *Ex situ* bags needed	[20]
ERBs with BSA	19.5	Low	Bringing cows to Mars is not feasible with current technology	This study [20],
ERBs with HSA	25.0	Low	Limited production. Potentially detrimental to crew wellbeing	This study
ERBs with HSA and urea	39.7	Low	Limited production. Potentially detrimental to crew wellbeing	This study
ERBs with synthetic spider silk	n/a	Low/Medium	Low technology readiness	This study
ERBs with natural spider silk	n/a	Low	Spiders are difficult to farm for silk	–

Other *in situ* human resources, such as hair and nails (keratin), dead skin cells (collagen), mucus, urine, and human feces, could also be exploited for their material properties on early extraterrestrial colonies. In this study, we explored the incorporation of urea — the most abundant constituent of human urine after water — into HSA-ERBs. As a protein denaturant, urea was hypothesized to affect the cohesive strength of HSA-ERBs since the proposed bonding mechanism is dependent on protein unfolding into a more thermodynamically stable β-sheet-rich confirmation. The incorporation of urea was found to increase the compressive strength of the HSA-ERBs by up to 300% (UCS of 39.7 MPa), while also increasing the density and light-atom content — which would likely improve the radiation-shielding potential of the materials [7]. The demonstration that frozen human feces does not produce effective knives recently won the 2020 Ig Nobel prize for Materials Science [22,23]; however, the low Martian surface temperatures (as low as −63 °C) and pressures could potentially make frozen or desiccated feces-based tools feasible — especially if combined as a composite material with Martian regolith and other human-derived feedstocks such HSA or urea. Unfortunately, due to health and safety concerns, we were unable to explore human feces-based ERBs in this study.

If HSA turns out to be unviable as a binder due to the physiological burden on the astronauts, mammalian serum albumin (MSA) from the plasma of cows, rabbits, goats, or other non-human species could also potentially be employed. We demonstrate that bovine serum albumin (BSA) has similar properties to HSA as a regolith binder, suggesting that the serum albumin from other mammals would also suffice. BSA-based ERBs have been explored extensively by D. Loftus and co-workers [20,[24], [25], [26], [27]]; our work suggests that HSA-based biocomposites would have analogous properties. The presence of non-human mammals on a Lunar or Martian mission would bring its own significant challenges, summarized in Table S1, but may also provide benefits such as a psychological boost to the astronauts, as well as a supplemental source of food (eggs, milk, meat, etc.) and materials (wool, leather, bone, etc.).

**Fig. 1:**
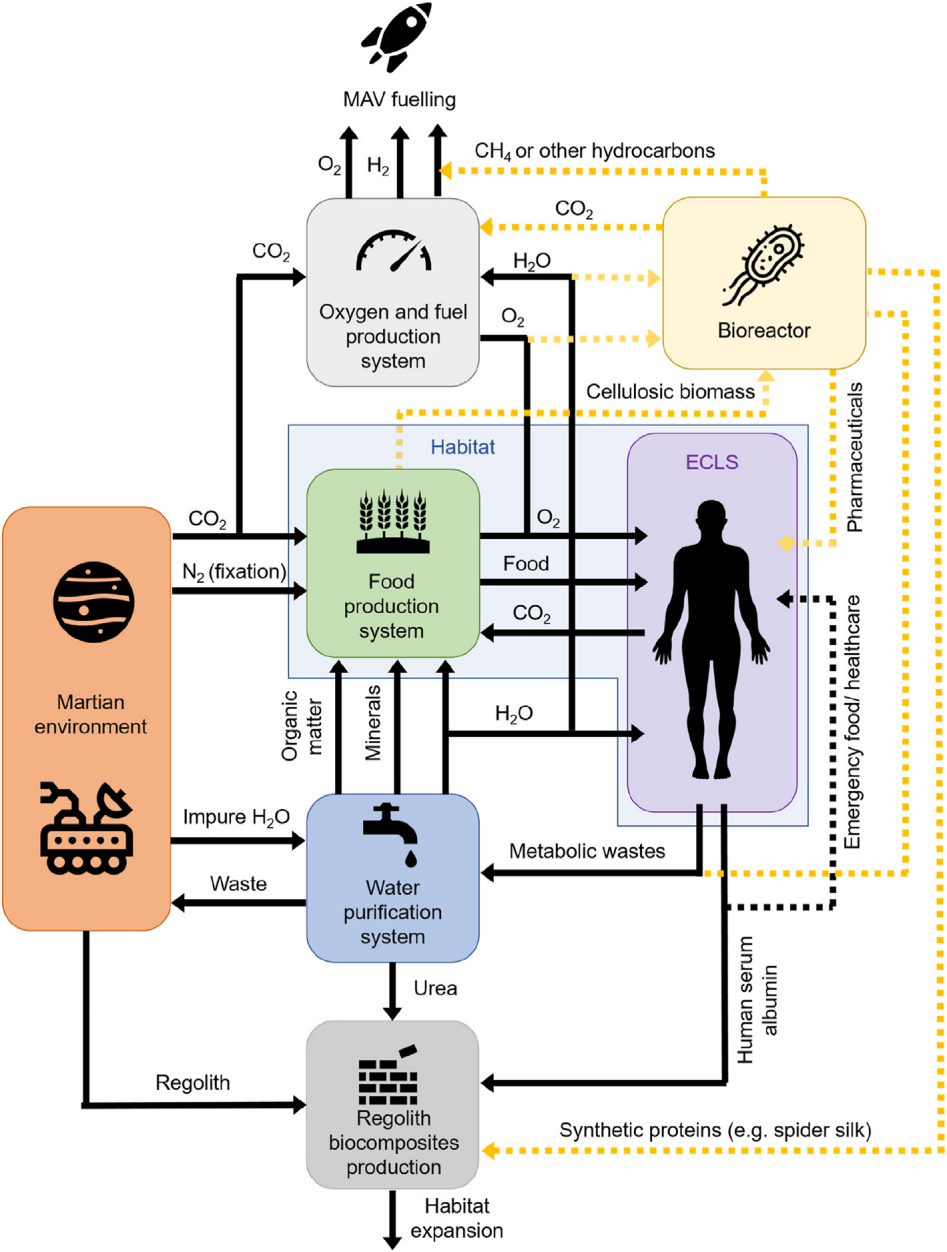
A hypothetical block diagram depicting how HSA could be produced *in vivo* from *in situ* resources available on Mars, and — technological advancements permitting — eventually supplemented or replaced with an ultra-high reliability self-contained bioreactor (dashed yellow arrows) that could have additional uses [28,30]. Abbreviations: Environmental Control and Life Support (ECLS), Mars Ascent Vehicle (MAV).

Synthetic (recombinant) proteins produced through microbial expression could be a more feasible option than bringing live animals into space. In this study, we also briefly investigated the feasibility of producing ERBs using a binder based on recombinant spider-silk — which could feasibly be produced on the surface of Mars if a self-contained ultrahigh reliability and minimal-waste bioreactor could be designed to operate on the planet [28]. Native spider silk could also potentially be produced, with spiders having already been extensively studied onboard the International Space Station (ISS) [29]. Fig. 1 depicts how HSA production could be incorporated with other critical systems needed for a closed-loop Martian habitat and eventually supplemented or superseded by a flexible bioreactor-based system [28,30,31]. The use of spider silk further supports the proposed mechanism of material cohesion being due to hydrogen bonding since the cohesive and adhesive strength of natural spider silk is attributed to the formation of densely hydrogen-bonded β-sheet networks [32].

## 2 Materials and methods

### 2.1 ERB fabrication procedure

In a typical procedure, 3 g of HSA (purchased from Sigma-Aldrich, lyophilized powder, >96% purity by agarose gel electrophoresis) was dissolved in 7 g of deionized (DI) water with gentle mixing at 40 °C to produce a 30 wt% solution. The density of water was taken as 1 g mL^−1^ and measured volumetrically. Note, higher mixing temperatures (>60 °C) will cause the protein to denature and form a gel. The solution would be used within 48 h and kept at 4 °C when not in use.

**Fig. 2:**
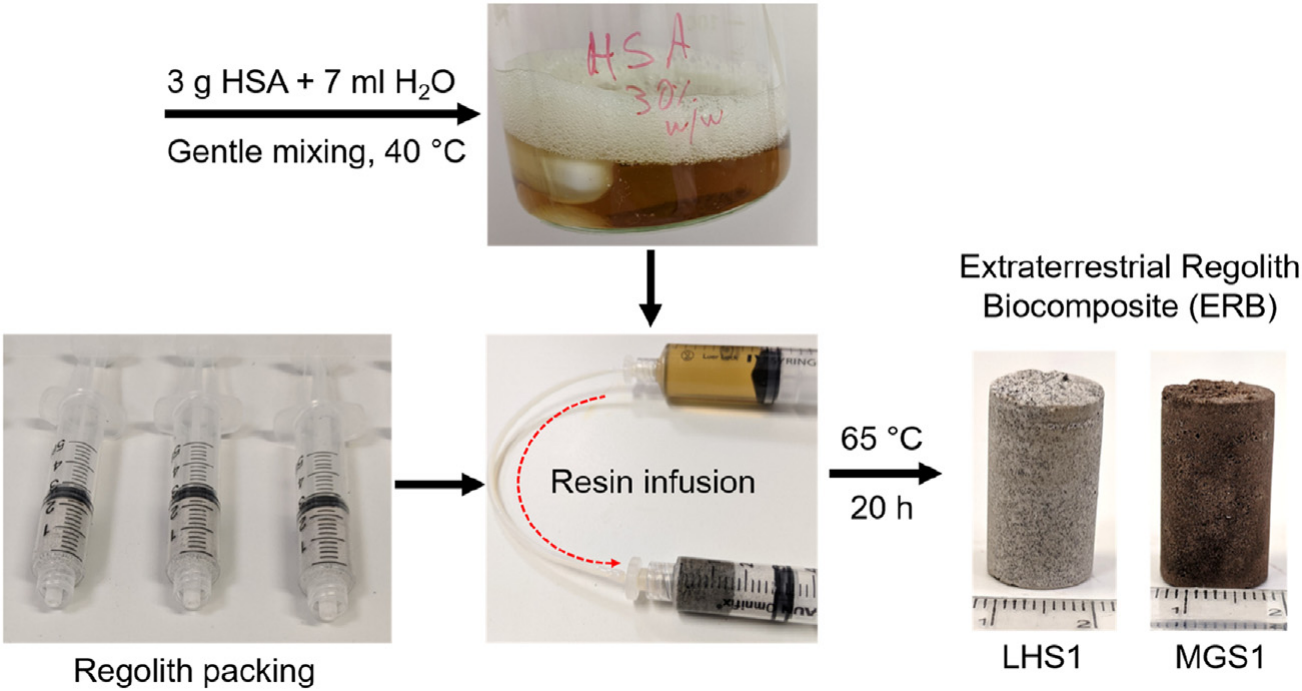
Scheme depicting the typical fabrication procedure for producing HSA-based ERBs.

Meanwhile, approximately 4 g of either Lunar Highlands Simulant 1 (LHS-1) or Martian Global Simulant 1 (MGS-1) (purchased from Exolith Lab, USA) were loaded into a 5 mL syringe (Omnifix® Luer-Lock, VWR International) and lightly packed with the syringe plunger manually. The precise composition of LHS-1 and MGS-1 is given in the SI. Following this, the HSA solution was infused into the pores of the regolith powder via another syringe, connected with Luer-lock attachments and PTFE tubing (Fig. 2). Note that small incisions were made at the end of the regolith-containing syringe with a razor blade to allow for pressure equalization. The masses of each empty syringe, syringe with regolith, and syringe with regolith and HSA solution were measured to allow for the calculation of binder-to-regolith mass ratio. The HSA-solution infused regolith syringes were then placed on a hot plate maintained at 65 °C overnight (*ca.* 20 h) with occasional removal of the supporting plastic syringe within the first 5 h to facilitate dehydration and hardening.

For the ERBs containing urea, the same procedure was employed, except solutions of urea (up to 3 M concentration) in DI water were employed as the solvent rather than DI water alone. The density of the urea solutions was taken as 1 g mL^−1^.

### 2.2 Uniaxial compression tests

To determine the UCS and stiffness of the ERBs we followed ASTM International Active Standard C39/C39M − 20 (Standard Test Method for Compressive Strength of Cylindrical Concrete Specimens). Briefly, the cylindrical ERB samples of 12.1 mm in diameter and roughly twice the length (∼24–30 mm) were subject to uniaxial compression tests using an Instron 5569 Series Universal Testing System (Instron Ltd., USA) that had been compliance corrected using a tungsten carbide disk. Before testing, the ERBs were sanded down manually with Emery paper until an even surface was achieved. The tests were conducted using a 5 kN load cell at a rate of 1 mm min^−1^ and continued until either sudden failure of the sample (40% drop in UCS) or if the sample was determined to have failed through visual inspection. The failure type was generally either type 2 or type 3 according to ASTM C39/C39M − 20 classification, with large vertical cracks propagating through the sample in line with the axis of compression (Fig. S1). The tests were conducted at ambient temperature and humidity, with sample ages ranging from a few weeks to about three months.

### 2.3 Circular dichroism

Circular Dichroism (CD) measurements were taken using a Chirascan V100 (Applied Photophysics, UK) through a method analogous to our previous publication on BSA and recombinant spider silk [17]. Briefly, 5 μL of HSA in DI water at 5 wt% concentration was drop-cast between two quartz substrates before a scan was taken between 190 nm and 300 nm, followed by subsequent scans every 60 min for 20 h. Note that higher HSA concentrations could not be employed as this would saturate the UV detector. The sample chamber was maintained at 25 °C, and the path length was approximated as 2 × 10^−4^ cm and assumed not to change over the duration of the measurement. The sample acquisition time was 12 min per spectrum (3s per point). For the thermal denaturation study, HSA in water at 0.4 wt% concentration was aliquoted into a quartz cuvette with a 0.1 mm path length. Higher HSA concentrations again could not be employed due to saturation of the UV detector. A CD scan was taken (between 190 nm and 300 nm) initially at 25 °C and then at every 10 °C intervals up to 85 °C, before declining at each 10 °C intervals back to 25 °C. Sample acquisition time was 5 min per spectrum (1 s per point) with a 2 min settling time between measurements to allow for thermal equilibration.

Protein secondary structure was determined using the online CD spectra analysis tool BeStSel, which fits experimental CD curves by the linear combination of the fixed basis components to calculate the relative proportion of the eight structural elements. Further details on the algorithms employed are available at http://bestsel.elte.hu/index.php or in Ref. [33].

### 2.4 Field-emission scanning electron microscopy (FE-SEM)

SEM images were taken on a Quanta 250 FE-SEM using secondary electron detection with an accelerating voltage of 8 kV and a working distance of 10 mm. Samples were adhered to aluminum studs with conductive carbon tape and sputter coated with Au (∼10 nm thickness) prior to imaging to enhance electrical conductivity.

### 2.5 Adhesive tests

Adhesion tests were conducted in an analogous manner to our previous publication, following ASTM International Active Standard D2919 [17]. Briefly, 5 μL of 30 wt% HSA in water was drop-cast onto a 2.6 cm width glass microscope slide (Fischer Scientific, Cat. No. 7101) before being laminated with another slide with an overlap of 0.5 cm — giving a total shear area of 1.3 cm^2^. Note that each glass slide was doubled up by adhesion with cyanoacrylate adhesive (Loctite® Superglue) to mitigate against substrate failure prior to adhesive failure [17]. The substrate-adhesive laminate was then compressed under mild pressure by the application of small (1.5 cm length) bulldog clips and left to bond under ambient conditions for a minimum of 30 h. The samples were subjected to single-lap shear adhesion tests under compression using an Instron 3340 Series Uniaxial Tensile Tester (Instron Ltd., USA) equipped with a 2 kN Load cell. The compression rate was 0.5 mm min^−1^, and the tests were conducted under ambient temperature and humidity. A cyanoacrylate adhesive (Loctite® superglue) was also tested under analogous conditions to have a side-by-side comparison of performance under the same test conditions.

### 2.6 Recombinant spider silk expression and purification

The recombinant spider silk protein (N-R_7_-C) used in this study was cloned and expressed as described by Finnigan et al. [34] Protein expression was performed using *E. coli* BL21 (DE3) cells cultured in Terrific Broth media supplemented with 100 μg μl^−1^ kanamycin. Transformed cultures were incubated at 37 °C and 180 rpm. Upon reaching an optical density at 600 nm of 0.8, cell cultures were induced by the addition of IPTG to a final concentration of 0.2 mM. Following induction, the incubation temperature was lowered to 20 °C for overnight expression. Cells were pelleted by centrifugation, resuspended in ice-cold buffer (25 mM Tris-HCl, 300 mM NaCl, 10 mM imidazole), and lysed by sonication on ice. Soluble protein was separated from insoluble cell debris by centrifugation. Recombinant silk protein was purified from the resulting supernatant by immobilized metal affinity chromatography using Ni-NTA resin and eluted using resuspension buffer containing 250 mM imidazole. Eluted protein was extensively dialyzed against 25 mM Tris-HCl, pH 8.0 at 4 °C to remove salt and imidazole. Aggregated silk proteins were removed via centrifugation. Purified silk protein was concentrated using VivaSpin ultrafiltration columns with a molecular weight cut-off of 10 kDa. Protein expression and purification were analyzed using SDS-PAGE (Fig. S2). The concentration of purified silk protein was determined in triplicate using a Nanodrop 2000 spectrophotometer. The A_280_ extinction coefficient and molecular weight of recombinant spider silk protein N-R_7_-C (21890 M^−1^ cm^−1^ and 34862.91 Da, respectively) were calculated using the ExPaSy ProtParam tool [35].

## 3 Results

### 3.1 Conceptualization and initial scoping experiments

Following the earlier work where we investigated the adhesion properties of BSA and recombinant spider silk on glass and other transparent substrates [17], it was hypothesized that BSA could also act as a binder for silicate-rich particulate materials such as sand or extraterrestrial regolith. A literature search revealed that extensive background work had already been undertaken by Loftus et al., who have evaluated the material properties of BSA-based ERBs (also termed biopolymer-bound soil composites, BSCs) in depth [20,[24], [25], [26], [27]].

The production of BSA *in situ* on a space mission would likely be problematic; either a cow would have to be transported along with the crew and their blood plasma periodically harvested — or the BSA could be produced recombinantly (i.e., synthetically) through a bioreactor [15]. If the latter option were to be employed, it would be more sensible to produce a protein with superior mechanical properties or otherwise greater utility than BSA, such as synthetic spider silk, squid ring teeth proteins, mussel adhesive proteins, or barnacle cement [15]. However, human-derived HSA has significant homology with bovine-derived BSA, and since humans would already be present on any crewed space flight, there would be no need to take additional animals such as cows onto the mission if the blood-plasma extraction method was to be employed. Therefore, we set out to investigate the material properties of HSA-based ERBs.

We employed a simple, custom-made setup for the fabrication of ERBs since complex, heavy equipment should ideally be avoided on a trip to Mars to minimize mission costs. It should therefore be highlighted that the use of more advanced fabrication techniques (e.g., hydraulic compression, vacuum-assisted resin infusion, etc.) would likely further improve material performance. Our fabrication method consisted of loosely packing approximately 2.4–3 cm^3^ of Lunar or Martian regolith simulant (LHS-1 and MGS-1, respectively) into a 5 cm^3^ disposable plastic syringe (Fig. 2.). Syringes were employed due to their consistent cross-sectional surface area (1.15 cm^2^), ability to connect with appropriate tubing for liquid binder infusion, and for their low-cost disposable nature. After regolith packing, a solution of HSA in water could be infused with the regolith by injection from another syringe.

The wet composites were weighed before and after infusion to allow the determination of the binder mass ratio. The wet composites were then heated to 65 °C to accelerate dehydration, with occasional removal of the supporting syringe tube to facilitate the escape of moisture. It should be noted that the methods used by Loftus et al. to produce BSA-based ERBs differed from our technique by employing a vacuum-assisted resin infusion method (VARIM) and room-temperature desiccation**.** [20].

### 3.2 Relationship between HSA-regolith mass ratio and ERB compressive strength

The compressive strength of aggregate-bound composite materials such as concrete or ERBs depends on numerous factors, such as binder-to-aggregate mass ratio, aggregate size and shape distribution, void size, and chemical composition [24,36,37]. Given the numerous challenges and constraints associated with extraterrestrial construction, pretreatment of extraterrestrial regolith should ideally be kept to a minimum. For instance, although Lunar and Martian regolith could be processed and refined to optimize properties such as chemical composition and particle size distribution, to do so *in situ* would necessitate processing equipment that would add to the mass, cost, and complexity of a mission. Therefore, in this study, we employed Lunar and Martian regolith simulants (LHS-1 and MGS-1, respectively) without additional processing. The precise composition of these simulants is given in the SI. One significant factor that could be easily and reliably controlled was the binder-to-aggregate mass ratio — which could be governed by initial protein solution concentration*.* [24].

This factor was, therefore, investigated by fabricating a number of HSA-ERBs with protein concentrations ranging between 15 and 37.5 wt%. A higher concentration of 40 wt% was attempted, but the solution was too viscous to infuse into the regolith. The binder-to-regolith mass ratio was determined by subtracting the dry regolith mass from the wet composite mass to determine the mass of HSA solution added, from which the dry mass of HSA could be determined based on the concentration of the solution. Compressive strength and stiffness (elastic modulus) were determined through uniaxial compression testing following ASTM guidelines (see Section 2 for details), with the data summarized in Table 2.

**Table 2:** HSA-ERBs prepared with different HSA protein concentrations: summary of binder-to-regolith mass ratio, compressive strength, and elastic modulus. The number in parenthesis indicates the number of samples tested.

Regolith type	HSA conc. (wt. %)	Binder-to-regolith mass ratio (%)	UCS (MPa)	Elastic modulus (MPa)
MGS-1	15	3.1 ± 0.1	1.9 ± 0.3 (5)	431 ± 111.8
MGS-1	20	5.4 ± 0.1	3.4 ± 0.4 (6)	662 ± 300
MGS-1	25	5.8 ± 0.2	5.8 ± 1.5 (6)	541 ± 224
MGS-1	30	8.8 ± 0.3	6.6 ± 1.8 (6)	1257 ± 390
MGS-1	35	8.1 ± 0.3	9.3 ± 1.2 (6)	968 ± 390
MGS-1	37.5	8.6 ± 0.3	6.4 ± 1.2 (6)	905 ± 343
LHS-1	15	3.7 ± 0.2	6.1 ± 1.7 (5)	236 ± 94
LHS-1	20	5.4 ± 0.4	9.4 ± 1.1 (6)	540 ± 158
LHS-1	25	6.1 ± 0.1	10.5 ± 3.0 (6)	1096 ± 474
LHS-1	30	8.0 ± 0.3	12.3 ± 2.2 (6)	1673 ± 890
LHS-1	35	8.6 ± 0.3	25.0 ± 3.1 (5)	1618 ± 479
LHS-1	37.5	10.8 ± 3	17.7 ± 6.8 (6)	1568 ± 578

**Fig. 3:**
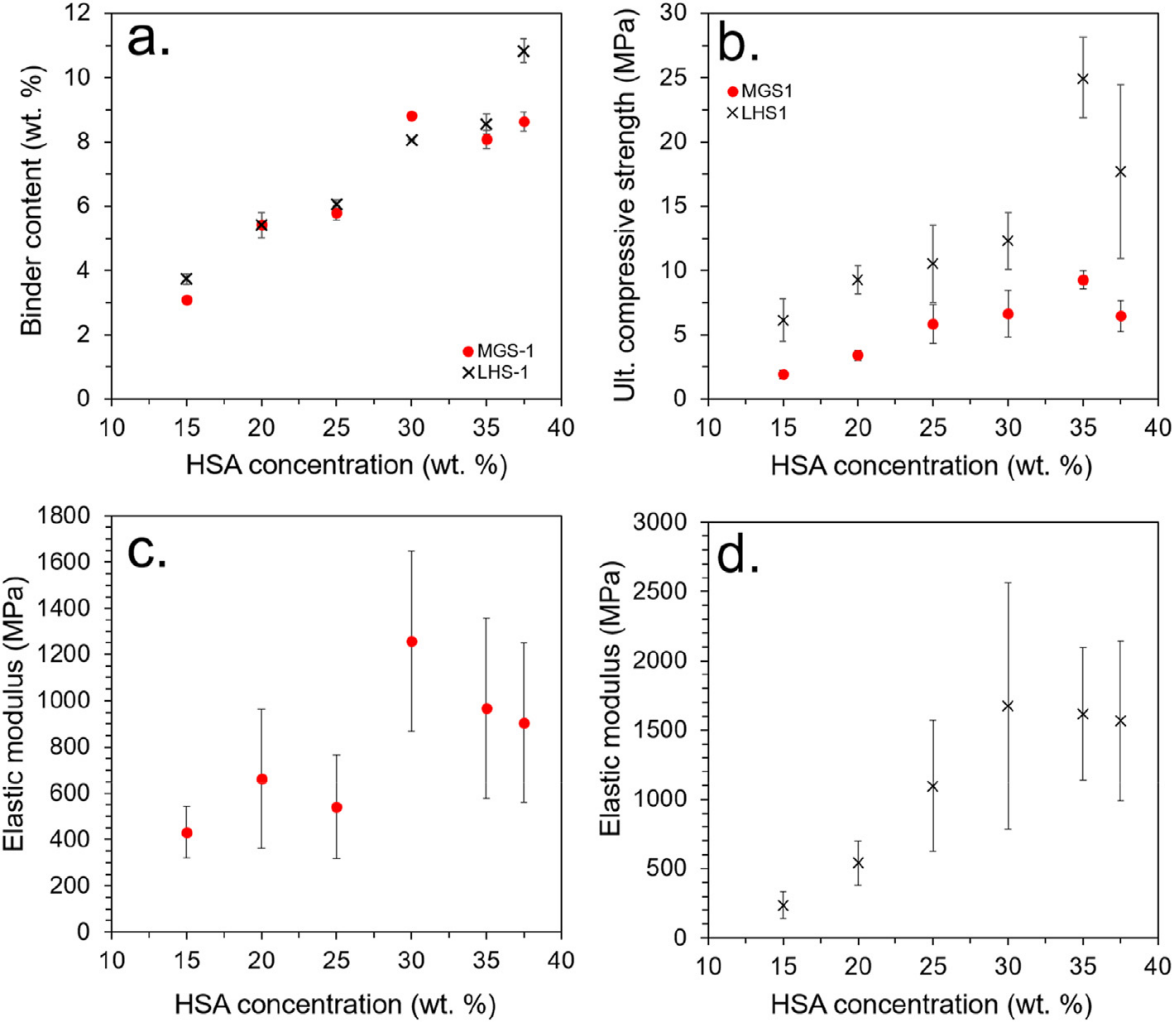
(a) Relationship between HSA solution concentration and calculated binder content. (b) Relationship between HSA solution concentration and UCS of the ERBs. (c) Relationship between elastic modulus under compression and HSA solution concentration for MGS-1 and (d) for LHS-1.

The data show a positive correlation between HSA concentration and calculated binder content (Fig. 3a). The data also show a clear positive correlation between HSA concentration and ultimate compressive strength (UCS) up to a concentration of 35 wt% (Fig. 3b), likely due to a generally increasing binder-to-mass ratio improving adhesion between particles. For both LHS-1 and MGS-1, the UCS dropped at the higher HSA concentration of 37.5 wt% — this was attributed to the higher viscosity of the solution, likely impeding impregnation into the smallest cavities and pores. A similar trend was also observed for the stiffness (i.e. the elastic modulus under compression) of the materials, which peaked at 30 wt% HSA (Fig. 3c and d). Visible light images of representative samples before and after compression tests, along with typical stress-strain profiles, are shown in Fig. S1. Our previous study on BSA-based adhesives conducted a rheology study on aqueous BSA solutions, which found that viscoelastic properties, including storage modulus, elastic modulus, and dynamic viscosity, all increased dramatically between a concentration of 30 and 40 wt% and were detrimental to adhesion [17].

LHS-1 displayed consistently higher UCSs than MGS-1, which was primarily attributed to the physical properties of the aggregate particles [7]. LHS-1 is significantly coarser than MGS-1, which is an extremely fine powder. Coarser particles facilitate a more even distribution of stresses within aggregate-based composite materials, which corresponds to a higher toleration of compressive forces. Optimization of MGS-1 particle size distribution (e.g., through sieving) would likely improve the compressive strength of the resulting ERBs, but to do so *in situ* would increase the mission complexity and cost.

In order to benchmark and compare against the previous findings of Loftus et al. [20,[24], [25], [26], [27]], BSA-based ERBs employing a BSA concentration of 30 wt% were also fabricated and tested. These displayed notably superior performance to HSA-based ERBs at the same concentration, with the LHS-1 based BSA-ERBs having a UCS of 19.5 ± 1.7 MPa and the MGS-1 based BSA-ERBs having a UCS of 12.0 ± 1.3 MPa. We note that BSA is significantly cheaper than HSA and so could be a useful model compound for future studies. Finally, we also tested a ready-mixed brick mortar (purchased from Wickes® Stockport, UK) as a further benchmark, where the UCS from six replicate measurements was found to be 6.1 ± 1.7 MPa — significantly lower than the ERBs.

### 3.3 Probing the bonding mechanism

Cement and other high-strength bonding materials such as epoxy resin and cyanoacrylate adhesives generally rely on the formation of covalently bonded crosslinked networks (e.g., silicate networks in cement) to harden and bind aggregate and other materials together. It is noteworthy then that the HSA employed in this study, as well as other protein-based binders in previous literature reports, should display such high bonding strengths without any apparent covalent crosslinking, relying exclusively on physical interactions for cohesion and adhesion [14,17]. The mechanism behind such non-covalent protein bonding is complex and has yet to be fully resolved, but likely involves a mixture of interactions, including entanglement, ionic-, hydrophilic-, and hydrophobic bonding [14]. We recently proposed a mechanism based on the dehydration-induced reorganization of protein secondary structure from a relatively α-helix rich conformation to a densely hydrogen-bonded supramolecular β-sheet network, supported by circular dichroism (CD) study [17]. This mechanism is analogous to the phase transformation of native spider silk from a concentrated liquid-crystalline solution (in the ampulla) to a β-sheet rich network (through the spinning duct), where extensive intermolecular hydrogen-bonding interactions contribute to the exceptional strength and toughness of spider silk [32,34,38,39]. Our previous work demonstrated remarkably similar changes to protein secondary structure when comparing BSA with recombinant spider silk over the course of adhesion, suggesting a similar underlying mechanism [17].

**Fig. 4:**
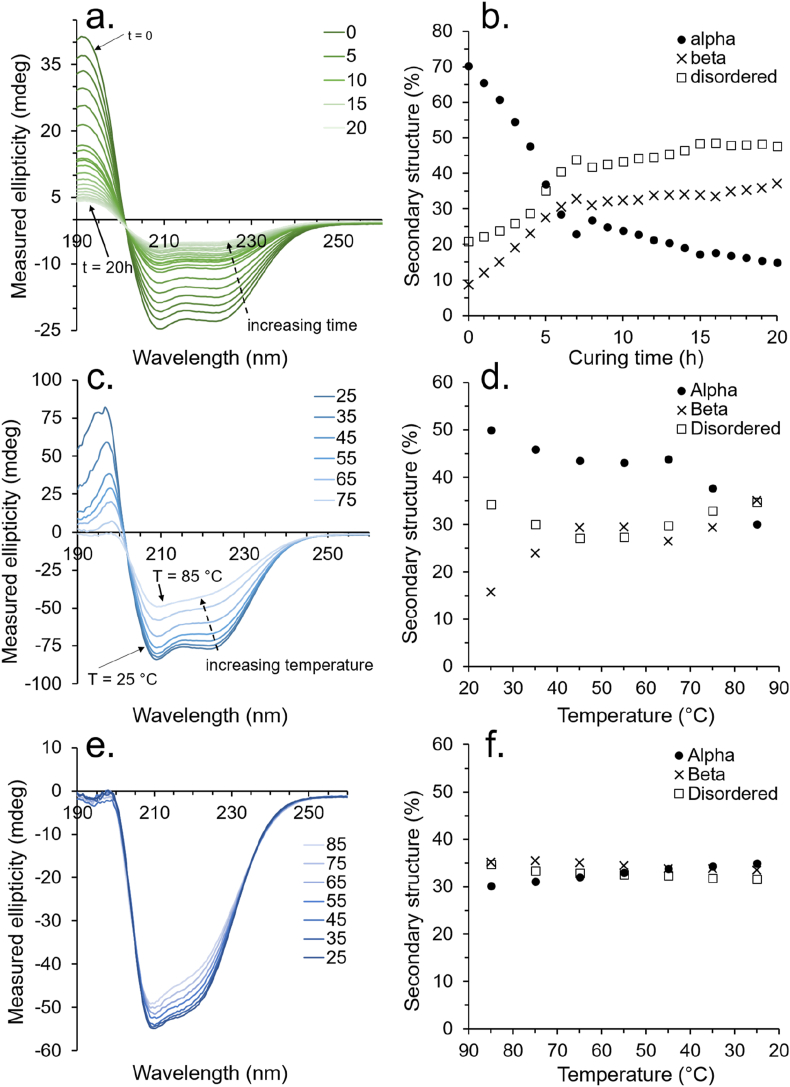
(a) CD spectra of a 5 wt% HSA solution laminated between two quartz substrates over a 20 h period with (b) corresponding changes to secondary structure. (c) CD spectra of a 0.1 wt% HSA solution from 25 °C to 85 °C with (d) corresponding changes to secondary structure. (e) CD spectra of the 0.1 wt% HSA solution from 85 °C to 25 °C with (f) corresponding changes to secondary structure.

Given the significant structural and functional homology between BSA and HSA [40,41], we hypothesized that an analogous bonding mechanism was occurring for HSA, as we had previously shown for BSA. For testing this hypothesis, a CD was employed on a thin film of 5 wt% HSA laminated between two quartz substrates, with measurements taken every hour over a 20 h period at 20 °C (see Section 2 for details). Concentrations above 5 wt% could not be employed as this would saturate the UV detector. After the 20 h period, the quartz substrates were strongly bonded and had to be soaked in water overnight to induce de-bonding. The obtained CD spectra are presented in Fig. 4a, with a breakdown of the secondary structural assignment given in Fig. 4b. A full breakdown of the secondary structure along with values for normalized root-mean-square deviation (NRMSD) to indicate the quality of data fitting is given in Table S2. The data show a clear change in the secondary structure from being initially relatively α-helix rich (70.3%) and β-sheet poor (8.9%) with a low proportion of disordered structure (21.0%) to having significantly less α-helix content (15.0%) but a higher content of β-sheets (37.3%) and disordered (47.7%) secondary structure after the 20 h adhesion period. These data support our hypothesis that the bonding mechanism is driven by a dehydration-induced change in protein secondary structure analogous to BSA and spider silk [17]. Further evidence for a glass-like transition state during the desiccation of BSA has also been published by G. Brownsey et al. [42] Interestingly, they suggest that at high concentrations BSA behaves like a colloidal suspension of globules — which is again analogous to how native spider silk behaves when stored as a high concentration liquid in the ampulla [32,38].

The thermal stability of HSA in the solution was also probed by CD spectroscopy. Here, a 0.4 wt% solution of HSA in water was aliquoted into a quartz cuvette with a path length of 0.1 mm. Again, higher concentrations could not be employed due to the saturation of the detector. CD measurements were taken at 10 °C intervals beginning at 25 °C up to 85 °C (Fig. 4c and d) before back down to 25 °C (Fig. 4e and f). The data show irreversible thermal denaturation of the HSA over the course of this heat treatment, occurring primarily between 65 and 75 °C, which is in line with the literature [43]. Interestingly, the degree of secondary structural change was far less pronounced than was observed for the dehydration-induced bonding — with total α-helix, β-sheet, and disordered secondary structure content all stabilizing in the region of 31–35%. A full breakdown of the secondary structure along with NRMSD is given in Table S3.

To investigate whether the process of denaturation was an important part of the bonding mechanism or whether denatured HSA would bond as effectively as non-denatured HSA, we intended to denature HSA prior to infusion with the regolith aggregate. This was, however, not possible since the thermally denatured HSA formed a gelatinous material that could not be infused into the aggregate. We conclude, therefore, that only non-denatured HSA can be employed due to appropriate viscoelastic properties — but it remains unclear if the process of denaturation is critical to the bonding mechanism.

FE-SEM was employed to visualize the microscopic bonding mechanism between HSA and aggregate particles (Fig. S3). The images appear to show ligament-like ties between particles, which is in concordance with the findings of Roedel et al. [20,25].

### 3.4 Incorporating urea into the formulation

Human urine will be produced in a significant quantity (0.8–2 L per crew member per day) on any long-term crewed space mission and will likely be fully recycled to maximize utility and avoid waste [31]. Aside from the recovery of water, which comprises 91–96% of urine [44], other metabolic wastes, including inorganic salts, urea, uric acid, and other organic compounds, will be recovered and recycled. Along with other human excreta, the primary use of processed urine will likely be as a fertilizer for plant growth [45,46].

The average person produces approximately 59 g of total urine solids per day, of which urea is the largest component at over 50% of the total [44]. Urea is also present in human sweat and tears [47,48], also predicted to be in high abundance on any Martian mission. Its relatively high abundance means that, ideally, urea should be employed for higher value applications than purely as a fertilizer, especially since atmospheric Martian N_2_ could be biologically fixed to make up for any shortfall in a closed human — plant nitrogen cycle [49].

Concentrated urea is a powerful protein denaturant and is routinely used in biological laboratories for this purpose [50]. Urea also forms strong hydrogen-bonded networks with itself and other compatible molecules [51]. Given that the HSA adhesion mechanism was attributed to protein denaturation (unfolding) and reorganization into a strongly hydrogen-bonded β-sheet rich conformation, we thought it would be interesting to investigate the effect of urea incorporation into the HSA formulation.

To do this, we employed solutions of urea in water as the HSA solvent rather than pure water. Relatively high HSA concentrations of 30 and 35 wt% were investigated since these had previously displayed the highest ERB compressive strengths (see section 3.2). Urea solutions of 1 M, 2 M, and 3 M concentrations were investigated to determine any trends with increasing concentration. A higher urea concentration of 4 M was also attempted, but this caused the HSA solutions to gel — likely due to a combination of partial protein denaturation and supersaturation. The combination of 35 wt% HSA and 3 M urea was too viscous for infusion with MGS-1. The marginally higher density of urea solutions compared to water (e.g., 1.03 g mL^−1^ for 3 M urea) were ignored and taken to be equal to the water in order to keep other factors (e.g. sample preparation procedure) consistent.

**Fig. 5:**
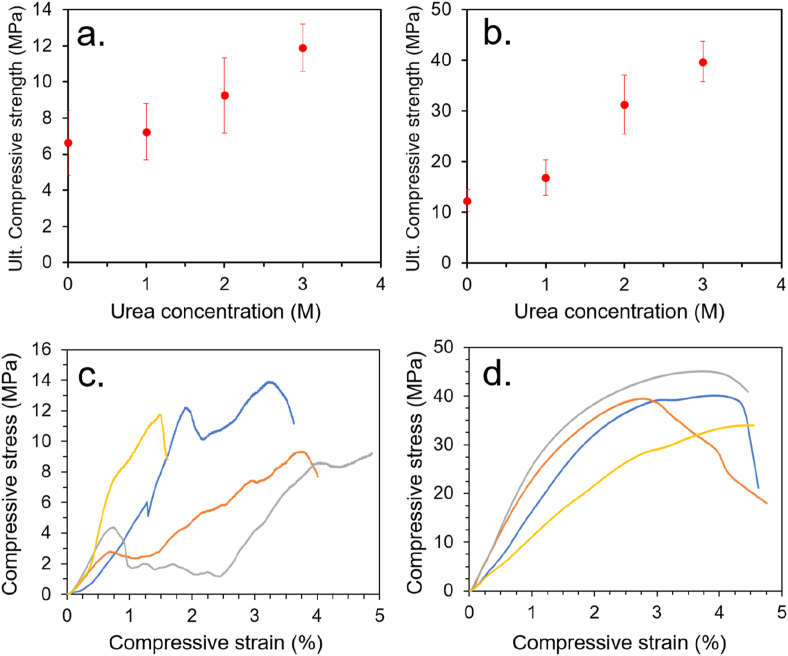
(a) Relationship between urea concentration and UCS for ERBs employing 30 wt% HSA with MGS-1 and (b) LHS-1. (c) Stress-strain curves EBRs employing 30 wt% HSA and 3 M urea with MGS-1 and (d) LHS-1.

A summary of the materials produced, along with their compressive strength and stiffness (elastic modulus), is given in Table 3. The UCSs of the materials were found to increase significantly with the incorporation of urea. For MGS-1 with 30 wt% HSA, the UCS increased by 179% with the use of 3 M urea compared to pure water. For MGS-1 with 35 wt% HSA, the UCS increased by 112% when 2 M urea was employed compared to pure water. For LHS-1 with 30 wt% HSA, the UCS increased by 324% when 3 M urea was employed compared to pure water — and by 149% for the HSA 35 wt% formulation with 2 M urea compared to water. For the 30 wt% HSA formulations with urea, the increase in compressive strength correlated well with increasing urea concentration (Fig. 5a and b). The trend was less pronounced with 35 wt% HSA, which may have been due to the relatively high viscosity of the solutions impeding infusion, resulting in voids that weakened the composites. Representative stress-strain curves for EBRs employing 30 wt% HSA and 3 M urea are given in Fig. 5c and d.

**Table 3:** Summary of HSA-ERBs prepared with 30 and 35 wt% HSA and various urea concentrations, including compressive strength and elastic modulus. The number in parenthesis indicates the number of samples tested.

Regolith type	HSA conc. (wt. %)	Urea conc. (M)	UCS (MPa)	Elastic modulus (MPa)
MGS-1	30	0	6.6 ± 1.8 (6)	1257 ± 390
MGS-1	30	1	7.2 ± 1.5 (4)	532 ± 167
MGS-1	30	2	9.2 ± 2.1 (4)	1586 ± 517
MGS-1	30	3	11.9 ± 1.3 (4)	939 ± 508
MGS-1	35	0	9.3 ± 2.1 (6)	968 ± 390
MGS-1	35	1	8.8 ± 2.2 (4)	1143 ± 593
MGS-1	35	2	10.4 ± 2.3 (3)	1540 ± 893
MGS-1	35	3	Too viscous	Too viscous
LHS-1	30	0	12.3 ± 2.2 (6)	1673 ± 890
LHS-1	30	1	16.8 ± 3.5 (4)	998 ± 337
LHS-1	30	2	31.2 ± 5.8 (4)	1916 ± 544
LHS-1	30	3	39.7 ± 3.9 (4)	1746 ± 354
LHS-1	35	0	25.0 ± 3.1 (6)	1618 ± 479
LHS-1	35	1	23.7 ± 3.3 (4)	4508 ± 3616
LHS-1	35	2	37.4 ± 2.2 (4)	1823 ± 627
LHS-1	35	3	29.5 ± 1.3 (4)	1239 ± 122

We hypothesize three primary mechanisms to account for the increase in USS from the incorporation of urea, namely: (1) that urea facilitates the unfolding of the HSA from an α-helix rich globular structure to the densely hydrogen-bonded β-sheet structure, (2) that urea promotes intramolecular hydrogen bonding between proteins and aggregate particles, and (3) that urea crystallizes out upon dehydration providing additional aggregate particles for the HSA to bind to. To investigate the proposed hypothesis, CD was employed on a HSA 5% formulation dissolved in 3 M urea in an analogous manner to the previous CD measurements. This again revealed significant changes to the CD profile over the course of the 24 h bonding period (Fig. S4); however, the secondary structure could not be determined due to poor peak fitting — likely due to interference from the presence of urea. A more in-depth study would therefore be needed to unpick the mechanism.

In addition to significantly increasing the compressive strength of the ERBs, the incorporation of urea would also increase the density and light-atom content of the materials. This would be expected to improve the radiation shielding potential of the ERBs — particularly neutrons generated by cosmic rays [7].

### 3.5 Investigating recombinant spider silk ERBs

Recombinant protein production has been employed for decades and is well known for the production of synthetic human insulin [52]. Recombinant proteins have also been explored for materials science applications, most notably the production of synthetic spider silk for its impressive material properties [15,34,[53], [54], [55]]. The versatile bioproduction of materials, fuels, fine chemicals, pharmaceuticals, and food on the surface of Mars could result in significant reductions in payload mass (between 26 and 85%) compared to conventional approaches [28,30], reducing mission cost. However, significant improvements in bioreactor efficiency, reliability, and recyclability of metabolic wastes will be needed before significant bioproduction on Mars will be viable [31], although significant advances in terrestrial bioproduction are expected in the near future [56,57].

As depicted in Fig. 1, HSA-based ERBs could eventually be supplemented or surpassed by recombinant protein production from versatile bioreactors, which may have a variety of other uses [28,30]. Although HSA can be produced recombinantly, proteins with superior properties or other beneficial features may as well be produced instead. For example, recombinant spider silk due to its exceptional mechanical strength and toughness [14,39,53], or squid ring teeth proteins for self-healing properties [58,59], among others [15].

**Fig. 6:**
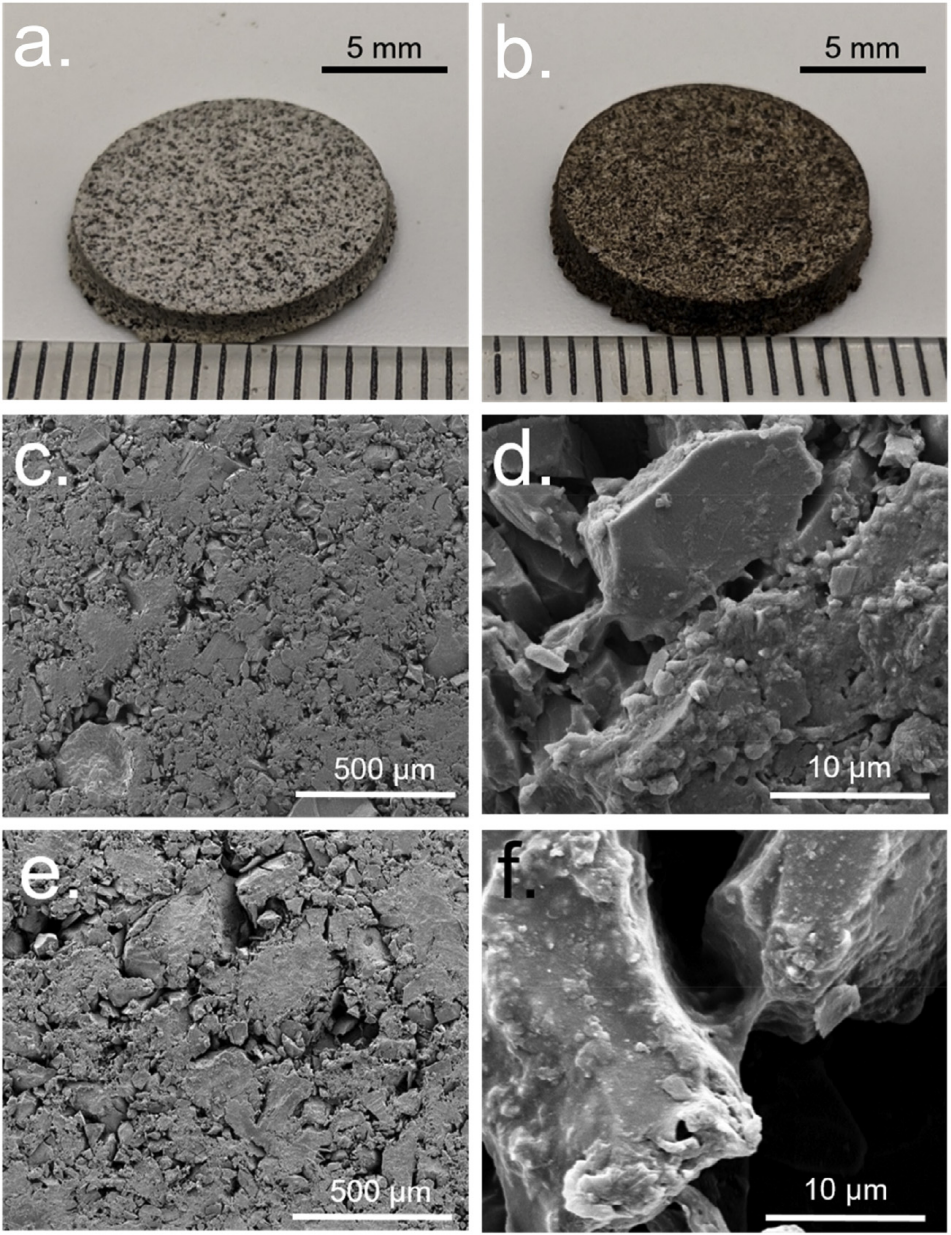
Visible light images of recombinant spider silk-based ERBs: (a) LHS-1 and (b) MGS-1. FE-SEM images of recombinant spider silk-based ERBs: (c) LHS-1 ×100 magnification, (d) LHS-1×5000 magnification (e) MGS-1×100 magnification, (f) MGS-1×5000 magnification.

To test whether, in principle, recombinant spider silk could be employed as a binder for extraterrestrial regolith — we produced and concentrated a small volume and fabricated ERBs with LHS-1 and MGS-1. Since the production of significant quantities of concentrated recombinant spider silk is difficult (the protein tends to spontaneously aggregate upon concentration), only a small volume of about 200 μL could be produced (average yield was 50 mg L^−1^). This was only enough to produce a single thin disk of about 2 mm in thickness and 13 mm in diameter for both LHS-1 and MGS-1 (Fig. 6a and b). This was not a suitable format for compression tests where much larger cylinders and several replicate samples are needed; therefore, we were not able to determine the mechanical properties in comparison to HSA or BSA. The experiment did, however, demonstrate that recombinant spider silk could, in principle, be employed to produce ERBs without any additional crosslinking agents. FE-SEM images also revealed similar ligament-like ties between the particles, as was observed with HSA (Fig. 6c–f).

### 3.6 Demonstrating the potential of HSA-ERBs for 3D-printing

Housing construction on Earth typically relies on a variety of materials with complex supply chains and laborious manual assembly by various specialists. This terrestrial approach is not viable in early Lunar or Martian colonies, which will require radically different construction techniques. Additive manufacturing (3D-printing) is regarded as a promising technique for extraterrestrial habitat construction since it can produce complex shapes and geometries, including hollow parts with internal support structures [[60], [61], [62]]. NASA’s Space Technology Mission Directorate recently concluded a Centennial Challenge competition with a focus on developing technologies for 3D-printed habitats, awarding over $2 million in total [5].

**Fig. 7:**
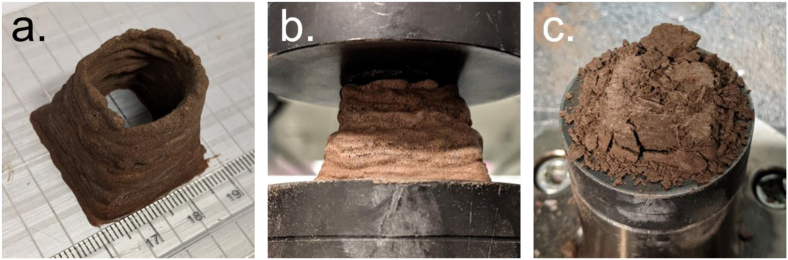
Visible light images of the 3D-printed HSA-ERB based on MGS-1. (a) after fabrication, (b) during compression testing, and (c) after compression testing.

Given the interest in 3D-printing for extraterrestrial habitat construction, we conducted a simple scoping experiment to determine if HSA-ERBs could feasibly be 3D-printed. Lacking appropriate 3D-printing equipment, we instead manually extruded a mixture of MGS-1 and HSA 15 wt% (3:1 mass ratio) out of a disposable 5 mL syringe in an approximate 25 × 25 mm square (wall thickness *ca.* 3 mm) onto an aluminum plate maintained at 65 °C. The HSA concentration of 15 wt% was selected due to its relatively low viscosity allowing for easier extrusion. After a 1–2 min resting period to allow for dehydration-induced hardening, further layers were added until a height of approximately 2 cm was achieved. The 3D-printed HSA-ERB was then left at 65 °C overnight to ensure thorough dehydration. Fig. 7a shows the completed 3D-printed construction after drying. The construction was subject to uniaxial compression until destruction to determine the strength of the construction (compression test set-up depicted in Fig. 7b and c), where it sustained an ultimate compressive force of 600 N - which is equivalent to a mass of 61 kg under Earth’s gravity, 162 kg on Mars or 370 kg on the moon (gravitational constants of 9.81, 3.71 and 1.62 N kg^−1^, respectively). The ultimate compressive strength of the construction was approximately 1.9 MPa but could potentially be improved with higher HSA concentrations and the incorporation of urea.

### 3.7 Adhesive performance of HSA on glass

Lunar and Martian regolith largely comprises silica (∼50%) and alumina (∼10%) meaning that the manufacture of glass-like ceramic materials would be possible through either sintering or melting appropriate ore or regolith [2,7,63,64]. The high energy cost of such processes (temperatures of 1200–1500 °C needed to melt regolith) may constrain the technique to the production of relatively small bricks, which would need to be further fused together by either heat, an adhesive, or a mortar for use as a construction material [7]. Although 3D-printing of regolith has been investigated, to date, the mechanical properties of the produced materials have been relatively low compared to other regolith binding methods, and significant equipment (i.e.*.* large mirrors) are needed [7]. The solar 3D-printing technique is also less feasible on the Martian surface than the lunar surface due to lower solar flux.

Here, we investigated the adhesive performance of a HSA-based solution on the glass to determine whether — in principle — HSA could be employed to adhere together glass-like materials such as sintered or melted regolith bricks. We have previously conducted an analogous investigation with BSA [17].

The results, presented in Table 4 along with a comparison of similar adhesives, found that HSA had an average ultimate shear strength (USS) of 5.2 ± 1.8 MPa based on seven replicate measurements (Fig. S5). As a direct comparison, a cyanoacrylate adhesive (Loctite® Superglue) was also tested under analogous conditions and had an average USS of 3.3 ± 1.2 MPa. Our previous study found that both BSA and recombinant spider silk had higher USS values at 8.53 ± 1.96 and 6.28 ± 1.09 MPa, respectively. Given the relatively small volumes of protein solution needed to conduct adhesive tests via this method (5 μL per sample), shear-lap adhesion tests such as these could be an efficient way to optimize formulations using difficult-to-produce recombinant proteins such as synthetic spider silk before scale-up to produce composite materials such as ERBs.

**Table 4:** Summary of the USS of HSA and BSA-based adhesives, recombinant spider silk-based adhesives, and (for comparison) some commercial adhesives on glass and aluminum determined by single-lap-joint shear tests. The number in parentheses indicates the number of replicate measurements.

Adhesive	Substrate	Ultimate shear stress (MPa)	Ref
HSA (30 wt%)	Borosilicate glass	5.2 ± 1.8 (7)	This work
Loctite® Superglue	Borosilicate glass	3.3 ± 1.2 (3)	This work
BSA (30 wt%)	Borosilicate glass	8.53 ± 1.96 (8)	[17]
BSA–ascorbic acid (10:1, 30% w/v)	Aluminum	2.8 ± 0.7	[65]
Spider silk (pH 8, 30 w/v %)	Borosilicate glass	6.28 ± 1.09 (8)	[17]
Spider silk (12% w/v)	Aluminum	1.16	[14]
Epoxy resin	Glass	14.4	[66]

## 4 Discussion

The remarkably high compressive strength of HSA-ERBs — which could be fabricated with minimal *ex situ* components without any heavy, malfunction-prone *in situ* binder production equipment — certainly warrants further investigation. We note that there is significant scope for improvement of material properties, too, with many factors such as curing temperature, compacting, and method of binder infusion having yet to be optimized. A significant amount of formulation optimization could also improve properties, such as pH and ionic conditions, or the inclusion of other *in vivo* substances such as feces, human hair, mucus, or other bodily fluids into the formulation.

**Fig. 8:**
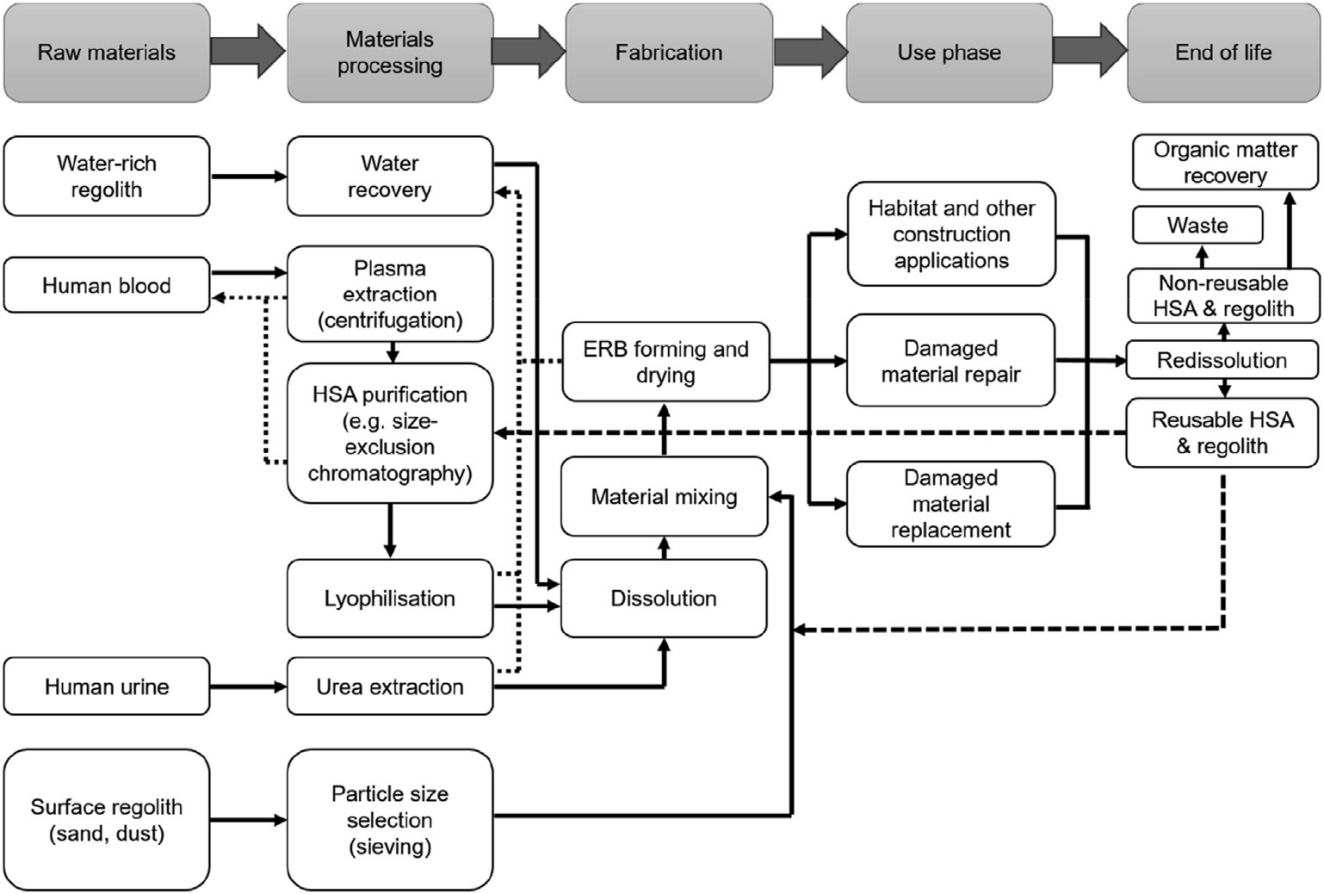
Life-cycle process flow diagram for HSA/Urea-based ERBs.

There is still a significant amount of uncertainty over the challenges associated with material processing for various proposed Martian binder technologies. *In situ* binders based on chemical and biochemical production will likely require significant downstream processing (e.g., purification, chemical modification, formulation, etc.), which will add extra complexity and risk of component failure. In contrast, the processing and use of HSA ought to be comparatively straightforward [18]. Fig. 8 shows a hypothetical life cycle process flow diagram depicting the important steps involved from raw materials to end-of-life, based on the sustainability assessment conducted by Loftus et al. [25] We note that HSA-ERBs should be fully recyclable at the end of life, either by dissolution and re-use of HSA as a binder — or recycling the organic matter for reclamation by the food production system (i.e. composting). Synthetic polymer/resin binders and Martian cement may be much more difficult to recycle at the end-of-life phase.

Even if HSA-ERBs turn out to be unviable for significant use on a Martian colony, understanding the potential uses and limitations of such materials could be critical in an emergency situation that requires flexibility and ingenuity to solve. The significant time delay between Earth and Mars, both in terms of logistical resupply (∼26 months) and communication (up to 44 min) [1], means that the ability for astronauts to devise solutions to novel threats and implement emergency repairs with the resources at hand will be critical to mission safety. The understanding that human blood plasma and urine, combined with regolith, can form a strong material could potentially solve a life-threatening emergency. As we have demonstrated, HSA-ERBs can also be 3D-printed using simple equipment (a syringe and 65 °C hot-plate) — which could be significant if a specific item, tool, or component needing a relatively high compressive strength needs to be produced in an emergency.

Finally, we note that there may be terrestrial applications from the pursuit of this technology. Concrete is, quite literally, a foundation material of the global economy and accounts for 8% of anthropogenic CO_2_ emissions [67]. These emissions are primarily from the thermal decomposition of calcium carbonate (CaCO_3_) to calcium oxide (CaO) — a primary component of cement. This chemical release of CO_2_, which accounts for over 50% of emissions, cannot be avoided through the use of renewable or nuclear energy sources, and thus, greener aggregate binders should be sought. The production of large quantities of biomanufactured proteins on earth has been a reality since synthetic insulin was mass-produced in 1982, and such proteins are now being commercially produced for various material applications. The application of structural proteins such as synthetic spider silk as an aggregate binder to replace cement could be feasible with future developments [25]; however, the extremely low cost and established nature of cement would make it difficult to displace without significant financial incentives. The use of naturally occurring plant-based biopolymers and bio-aggregates may be a more feasible and sustainable route forward [16]. A detailed discussion of additional practical considerations and a comparison between different extraterrestrial habitat construction technologies is presented the Supplementary Information and in the Supplementary Table S1.

## 5 Conclusions

The sweat and blood of astronauts, metaphorically speaking, has always been a critical prerequisite of any crewed mission to Mars. In this study, we propose that their literal blood may have an important secondary application in addition to its primary purpose: as a binder for extraterrestrial regolith to produce concrete-like biocomposites (ERBs). With ultimate compressive strengths as high as 25.0 MPa — or up to 39.7 MPa with the incorporation of urea — the produced ERBs can exceed the compressive strength of standard terrestrial concrete (typically 20–32 MPa) while having significant scope for further optimization.

Unlike other proposed *in situ* binder production technologies, this method circumvents the need for heavy and malfunction-prone binder production equipment (along with spare parts) needing to be delivered to the Martian surface. According to our calculations, a liter of human blood plasma contains enough HSA to produce about 300 g of ERB. If donated twice a week, each astronaut could produce about 2.5 kg of ERB per month — which is about the mass of a standard red clay brick. If employed as a mortar to bind together sandbags or bricks made from sintered or cast Martian regolith, our calculations suggest that each astronaut — over the course of a Martian mission — could produce enough additional habitat space to support another astronaut, potentially allowing the steady expansion of an early Martian colony.

Significant further investigation is still needed to determine the feasibility of the concept, particularly fatigue and durability evaluation under simulated Martian conditions (extreme thermal cycling, low pressures, high radiation, etc.) and the long-term health effects of continuous plasma donation in a reduced gravity environment. The quantity of HSA that could feasibly be extracted from astronauts without affecting their health would also need to be determined experimentally, and alternative options (e.g., use of plant-based proteins) should be explored. Despite this, we believe that HSA-ERBs could potentially have a significant role in a nascent Martian colony but will eventually be superseded by versatile bioreactors or other technologies as they mature.

## Credit author statement

Aled Roberts: Conceptualization; Data curation; Formal analysis; Investigation; Methodology; Writing - original draft, Dominic Whittall: Investigation; Methodology; Writing - review and editing, Rainer Breitling: Project administration; Resources; Supervision; Writing - review and editing, Eriko Takano: Project administration; Resources; Supervision; Writing - review & editing, Jonny Blaker: Project administration; Resources; Supervision; Writing - review and editing, Sam Hay: Project administration; Resources; Supervision; Writing - review and editing, Nigel Scrutton: Conceptualization; Funding acquisition; Project administration; Resources; Supervision; Writing - review and editing.

## Declaration of competing interest

The authors declare that they have no known competing financial interests or personal relationships that could have appeared to influence the work reported in this paper.
