# Supplementary material for: Blood, sweat, and tears: extraterrestrial regolith biocomposites with in vivo binders
Source: Mater Today Bio. 2021 Sep 10;12:100136. doi: 10.1016/j.mtbio.2021.100136 (PMC8463914; doi:10.1016/j.mtbio.2021.100136)
Supplement: Multimedia component 1 [file mmc1.pdf]

Supplementary Information

Blood, sweat and tears: extraterrestrial regolith biocomposites with *in vivo* binders

Aled D Roberts[a†], Dominic Whittall[a], Rainer Breitling[a], Eriko Takano[a†], Jonny J Blaker[b], Sam Hay[a] and Nigel S Scrutton[a†]

a. Manchester Institute of Biotechnology and Department of Chemistry, The University of Manchester, Manchester M1 7DN, UK

b. Bio-Active Materials Group, School of Materials, The University of Manchester, Manchester, Manchester M1 2PG, UK

[†] EPSRC/BBSRC Future Biomanufacturing Research Hub

Corresponding author: Professor Nigel S. Scrutton, Future Biomanufacturing Research Hub, Manchester Institute of Biotechnology, 131 Princess St, Manchester M1 7DN, UK; Tel: +44 (0) 161 306 5152, E-mail: Nigel.Scrutton@manchester.ac.uk

A discussion on the potential merits and drawbacks of the HSA-regolith binder concept

Effect on crew health

The main issue we foresee with the concept presented in this paper is the detrimental physiological and psychological effect that long-term HSA extraction will likely have on crew health and wellbeing, which would be the top priority of any mission. Space flight is already taxing on the human body, with numerous health issues – including muscle, bone mass and eyesight degradation – arising from living in microgravity with elevated stress and radiation exposure.[1,2] A procedure that further contributes to the degradation of crew health would likely be completely unacceptable to any space agency or private company. The health effects of long-term HSA extraction in a reduced gravity environment would therefore need to be thoroughly investigated before the technique could be employed in practice. The authors doubt that HSA extraction will be tolerable under microgravity due to concerns over crew health; however, the strength of gravity on Mars (62% lower than Earth's) could make HSA extraction possible without significant heath deterioration – although this would need to be confirmed experimentally.

Need for an initial habitat

Another significant issue with this concept is that habitat construction using this method can only occur once a crew has already landed on Mars and would take months to fully deploy, meaning a pre-built habitat complete with radiation protection will already have to exist for the first crew at least. Construction using HSA-ERBs could therefore only be employed for gradual habitat expansion or repairs. Other proposed technologies could feasibly be employed using robotic rovers and automated systems to construct a habitat prior to the arrival of the first humans.

Fire safety consideration

Fire safety has been noted as a high priority for extraterrestrial missions, which reduces the appeal of many synthetic polymer/resin binders, which are often volatile and flammable prior to curing. High temperature *in-situ* regolith sintering or polymer synthesis equipment may also pose a fire risk if housed within an O_2-rich crew habitat. HSA is non-flammable and does not require high-temperature processing so does not have this risk, which is a notable merit.

Toxicity consideration

The toxicity of many synthetic binders, such as epoxy resin adhesives, should also be considered. A leak of a volatile toxic compound within the environmental and life support (ECLS) system could be detrimental to the survival of the crew, whereas HSA is non-toxic and non-volatile. Epoxy resin composites might, however, achieve superior performance characteristics than protein-based binders.

Crew diet consideration

Crew diets will need to be supplemented with additional protein, calories and water to make up for the deficit arising from HSA extraction. However, we do not regard this as a significant issue since any long-term mission ought to have significant overcapacity in food and water production for redundancy – meaning a surplus of food and water will be produced under normal conditions. If an unforeseen problem reduces the amount of food or water that can be produced (e.g., crop disease or death), HSA extraction could be reduced or halted allowing astronauts to lower food intake to conserve supplies.

Demand for other resources

Energy, water and other resource consumption should also be considered when evaluating the merits of an *in-situ* binder. Many of the currently proposed methods for regolith binding – such as *in-situ* synthetic polymer/resin binders, Martian cement production or regolith sintering or melting – require large amounts of energy, water, oxygen and/or hydrogen. Demand for these resources will compete with other mission critical systems and also necessitate extra equipment for obtaining larger quantities of such resources (e.g., extra solar panels for energy

production, additional water extraction or water-splitting equipment, etc.). As discussed above, HSA production only requires a higher food and water intake by the crew, which will already be possible due to an over-production of food for contingency.

Raw material locality

Unlike most proposed cement-based Martian construction methods, HSA binders will be – by definition – produced on-site, whereas deposits of proposed Martian cement precursors can be geographically sparse which would constrain the location of a colony and possibly limit access to other important resources such as water deposits.[3] Mining, transportation, purification, milling and high-temperature calcination of mineral deposits such as gypsum or bassanite for Martian cement production would also necessitate heavy, energy intensive and malfunction-prone processing equipment.[3] The consumption of water, a relatively scarce Martian resource, for the curing of cement-based materials would also compete with other life-support systems such as food and oxygen production. The water employed for HSA-based binders, on the other hand, would be recoverable.

Raw material processing

The exact purity requirement for HSA has not been explored in this study, but Loftus *et al.* found that relatively impure bovine blood plasma could be employed as an effective binder rather than high-purity BSA.[4] HSA extraction and purification equipment will have to be an additional mission consideration; numerous possible purification methods exist and the most appropriate for a Mars mission would need to be selected.[5] Size- or steric-exclusion chromatography could be appropriate and allow non-albumin components of blood to be returned to the astronauts intravenously, minimising physiological stress.

Microbial degradation

HSA-ERBs could also be subject to microbial degradation over time; however, the inhospitable Martian environment (extremely cold, dry and low atmospheric pressure) would make this unlikely. We note that BSA and HSA-ERBs produced over a year ago and kept under ambient

conditions on Earth have not displayed any evidence of microbial decomposition, nor any other kind of visible degradation.

Material production limitations

Another major drawback of this concept is that the rate of HSA production will be limited by the number of crew. Whereas other theoretical *in-situ* binder production techniques could be scaled up by the transport of additional equipment to Mars, increasing HSA production would require additional astronauts. We calculate that approximately 550 kg of high-strength HSA-ERB could be produced over the course of a two-year (72 week) mission on the surface of Mars by a crew of six astronauts, assuming 1.2 litres of plasma is harvested twice a week – the upper end of WHO guidelines (calculations given below).[6] This rate of production is evidently too low for use as a stand-alone construction material, where is has been estimated that 24–144 tonnes of 3D printer feedstock would need to be produced on or shipped to Mars to support a 6-crew mission.[7] However, if employed as a mortar for bricks produced from sintered or melted regolith or sandbags produced *ex situ* and filled with regolith,[8–10] our calculations suggest that each crew member could, over the course of a mission, produce enough HSA to expand the habitat sufficiently to support one additional crew member. This could allow the steady expansion of an early Martian colony, especially since less launch mass will need to be dedicated to binder fabrication equipment and associated spare parts.[11]

Related alternative technologies

If HSA is deemed unfeasible as a binder for extraterrestrial regolith, it would also be possible to extract and employ serum albumin from the blood of other mammals given the homology of the proteins.[12] Animal husbandry in space and on Mars will come with its own significant challenges, summarised in Table S1, but would also have some advantages such as efficient recycling of cellulosic biomass, a source of other materials and foodstuffs (wool, milk, leather etc.) and a psychological boost for the crew. The animals could also be employed for other experiments. Albumins are also produced by pulses such as peas, which may be a more

convenient source than mammalian blood. However, pulse-derived albumins would require additional purification and processing, and are produced at a slower rate than serum albumins. We were unable to test pulse-derived albumins in this study due to lack of a high-purity commercial source.

As noted in section 3.5 and Figure 1., versatile bioreactors could be employed to produce recombinant HSA or other proteins with superior adhesion properties such as spider silk. Bioreactors will add a significant volume (50–2000 L) and mass to any Martian mission, and will require redundancy and spare parts, but could reduce payload mass by between 26–85 % compared to conventional approaches and have auxiliary uses.[7,13] Bioproduction technology still requires significant development before it could be employed for such applications with high enough reliability and efficiency, but as the technology matures – ERBs from synthetic proteins could supplement or replace HSA-based ERBs on Mars. We note that the concentration of HSA in human blood, at about between 35–50 g L^{-1},[14] is significantly higher than what is currently obtainable for similarly-sized proteins produced in contemporary bioreactors (typically <1 g L^{-1}), and contemporary bioreactors produce significant organic waste which would need to be processed.

Other applications

HSA extracted from the crew could have some other beneficial applications besides habitat construction. BSA is currently employed as a tissue adhesive,[15] so it is likely that HSA could be employed in an analogous manor. HSA could also be re-introduced into crew members *via* intravenous (IV) therapy to treat a variety of clinical conditions, including restoration of blood volume, treatment of shock, acute burns and other conditions associated with hypervolemia.[5] In the event of a serious failure in the food production system and its back-up, previously extracted HSA could be consumed in an emergency.

Back-of-the-envelope calculations on ERB production

The WHO advises a maximum blood plasma donation rate of twice a week and a volume up to 1.2 L per donation.[6] The concentration of HSA in the blood plasma of healthy adults is 35 – 50 g L^{-1}.[14] To calculate the total mass of HSA that can be produced over a mission period, the following equation was employed:

$$M_{HSA} = N_{crew} * D_{week} * N_{week} * C * V$$

Where M_{HSA} is the total mass of HSA produced over a mission period, N_{crew} is the number of crew on the mission, D_{week} is the number of donations per week, N_{week} is the mission duration in weeks, C is the concentration of HSA in blood plasma and V is the volume per donation. For a crew of six astronauts on a 72-week mission, assuming two 1.2 L donations per week and an average HSA concentration of 42.5 g L^{-1}, the total mass of HSA produced would be 44.1 kg. The total mass of ERB that could be produced (M_{ERB}) would depend on the HSA–regolith mass ratio, which we found ranges between 3.1 and 10.8 % (see Table 1). M_{ERB} could be calculated using the following equation:

$$M_{ERB} = \frac{M_{HSA}}{\theta_{ERB}} * 100$$

Where θ_{ERB} is the HSA regolith mass ratio in wt. %. Assuming that relatively high compressive strength ERBs would be produced, having a θ_{ERB} of 8 wt. %, a total of 550 kg of ERB could be produced over the course of a 72-week, six-crew mission to Mars.

Although M_{ERB} could be increased in various ways, such as increasing the frequency of plasma harvesting or reducing the HSA to regolith mass ratio, the rate of production is likely too low to produce significant structures comprised entirely of HSA-based ERBs. However, the use of regolith-filled sandbags or thermally sintered bricks combined with a suitable internal liner has been suggested as the most viable method for Mars construction.[9] If HSA-ERBs were combined with such a method, for instance as a mortar to bind together sandbags or sintered bricks, then the amount of construction material would go much further.

The recommended habitat volume per crew member is 120 m^3 for long duration (>4 months) missions, which would require about 24 metric tonnes of construction material.[7] Since each crew member could produce about approximately 91 kg of high-strength ERB – provided a brick-to-mortar mass ratio of about 261:1 could be achieved (i.e. through producing large bricks/sandbags and using minimal HSA-ERB mortar), then each crew member could, in principle, produce enough additional habitat space to support an additional future crew member. We should emphasise, however, that these calculations assume perfect extraction and replenishment of HSA which may not be feasible in space and the true rate of HSA extraction, and any losses through processing or storage, would need to be determined experimentally.

LHS-1 information and composition[16]

Reference material: Average lunar highlands

Bulk density (uncompressed): 1.30 g cm^{-3}

Mean particle size: 60 µm

Median particle size: 50 µm

Particle size range: <0.04 - 400 µm

Minerology (wt.%): Anorthosite (74.4), Glass-rich basalt (24.7), Ilmenite (0.4), Olivine (0.3), Pyroxene (0.2)

Bulk chemistry (wt.%): SiO_2 (48.1), Al_2O_3 (25.8) CaO (18.4), Fe_2O_3 (3.7), K_2O (0.7), MgO (0.3), MnO (0.1), P_2O_5 (1.0), TiO_2 (1.1), SO_3 (0.3), Cl (0.4), SrO (0.1)

MGS-1 information and composition[17]

Reference material: Rocknest soil

Bulk density (uncompressed): 1.29 g cm^{-3}

Particle size range: <0.04 - 600 µm

Minerology (wt.%): Plagioclase (27.1), Basaltic glass (22.9), Pyroxene (20.3), Olivine (13.7), Magnesium sulfate (4.0), Ferrihydrite (3.5), Hydrated silica (3.0), Magnetite (1.9), Anhydrite (1.7), Iron carbonate (1.4), Hematite (0.5)

Bulk chemistry (wt.%): SiO_2 (45.57), TiO_2 (0.30), Al_2O_3 (9.43), Cr_2O_3 (0.12), FeO_x (16.85), MgO (16.50), CaO (4.03), Na_2O (3.66), K_2O (0.43), P_2O_5 (0.37), SO_3 (2.63), MnO (0.1)

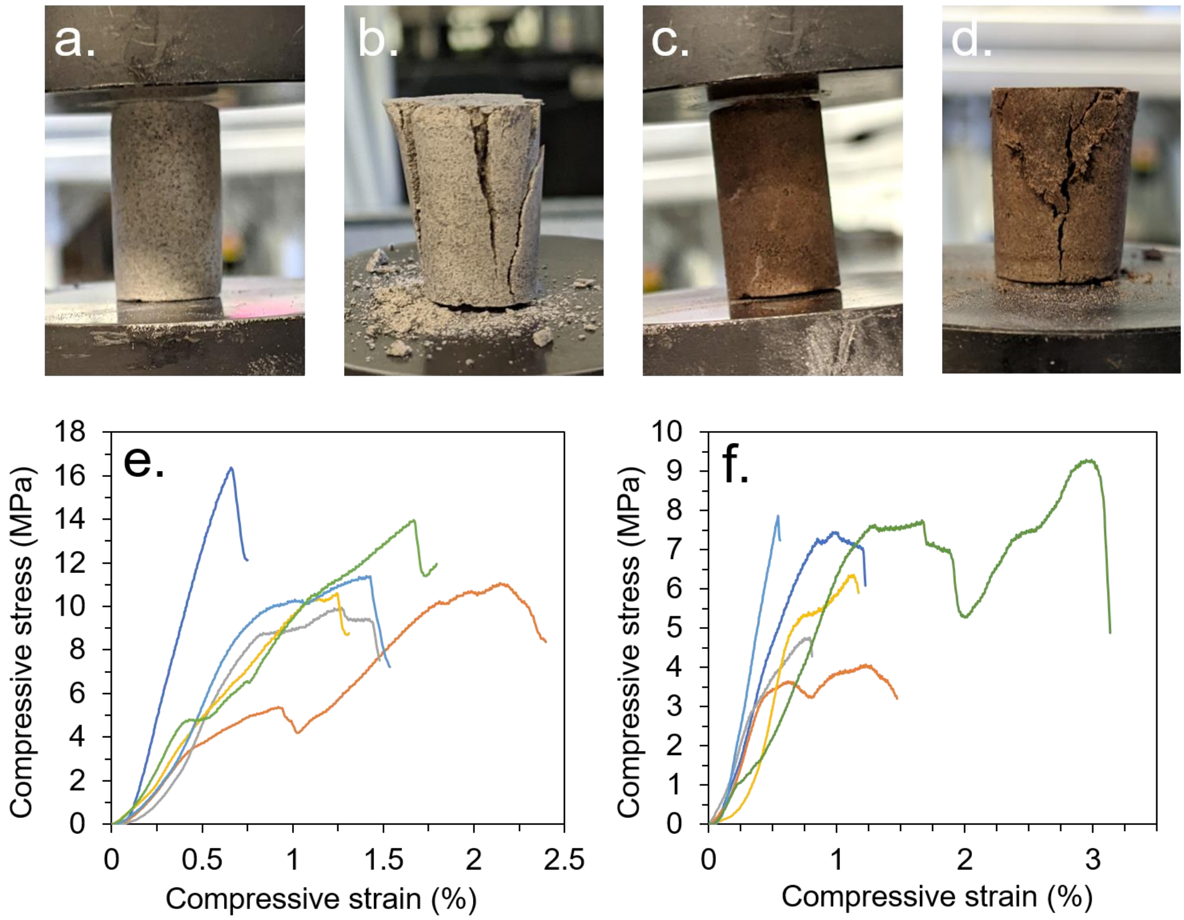

Figure S1. Visible light images of representative HSA-ERBs undergoing uniaxial compression tests. a) LHS-based ERB before and b) after compression test. c) MGS1-based ERB before and d) after compression test. e) stress-strain profiles ERBs prepared with LHS1 and 30% HSA, and f) MGS1 and 30% HSA.

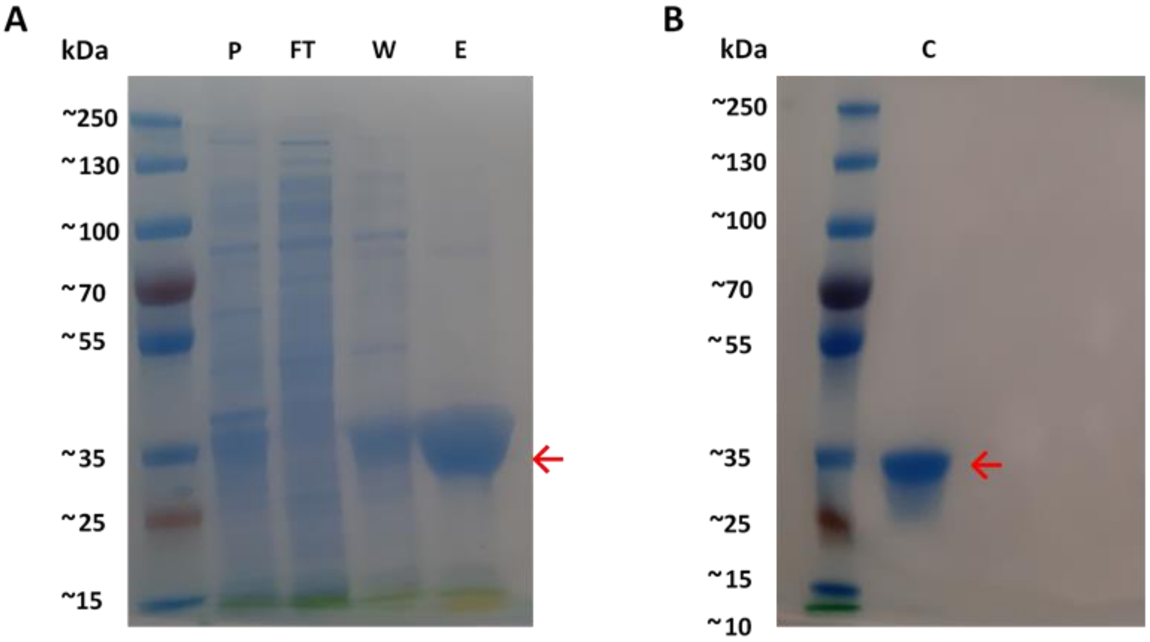

Figure S2. Expression and purification of recombinant spider silk protein N-R_7-C. A) Samples of the insoluble pellet (P); flow through (FT); wash (W) and elution (E) fractions following cell lysis and subsequent purification by Ni affinity chromatography analysed via SDS-PAGE. B) Sample of silk protein used in study following concentration (C), analysed using SDS-PAGE. Successful over-expression and purification of recombinant spider silk protein N-R_7-C is shown by bands at the expected molecular weight (~35 kDa) indicated by red arrows.

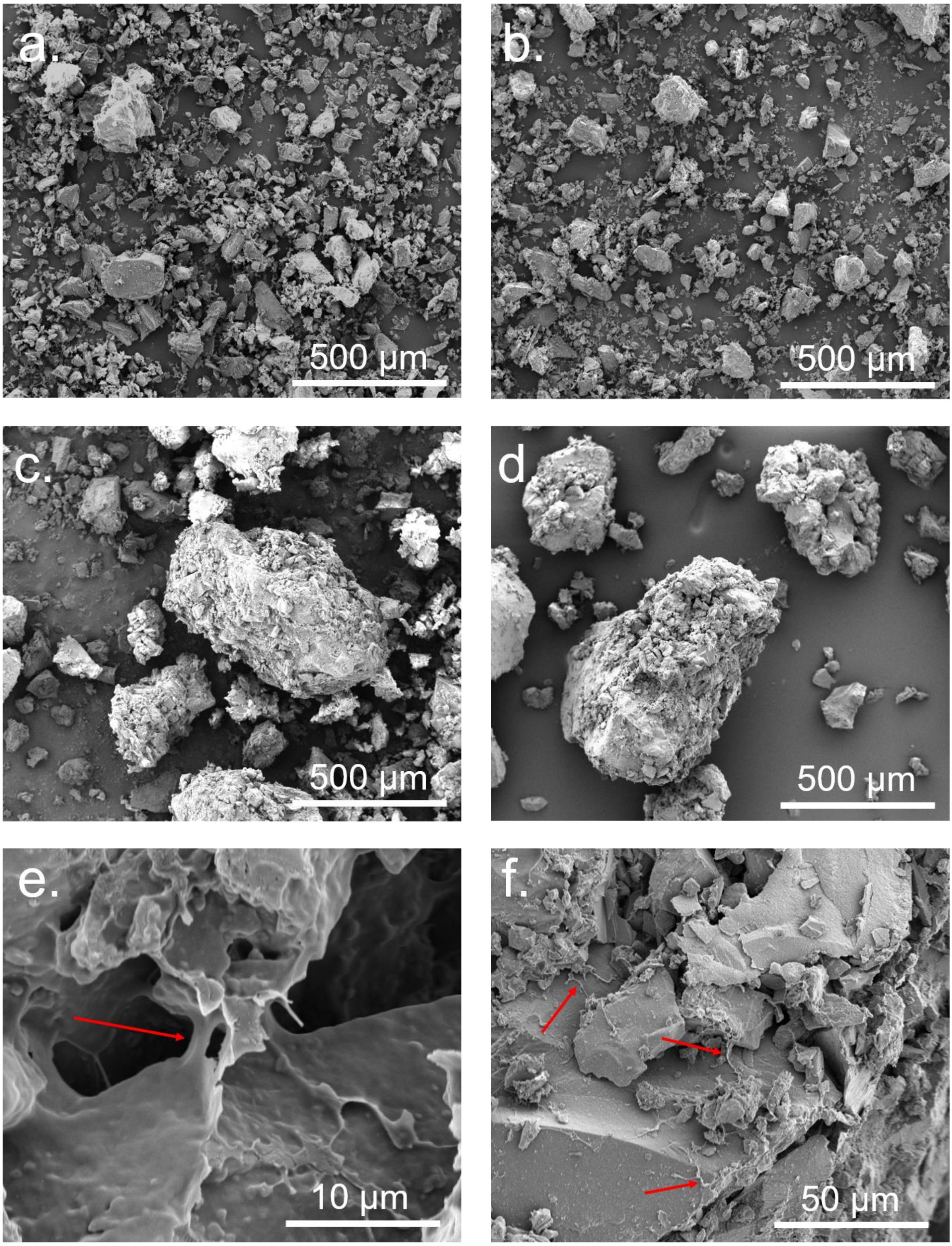

Figure S3. FE-SEM images showing a) LHS1 and b) MGS1 at 100x magnification as supplied by the manufacturer. c) LHS1-ERB and d) MGS1-ERB particles based on a 30 wt% HSA binder at 100x magnification. e) The same LHS1-ERB and d) MGS1-ERB at higher magnifications with ligament-like bonds highlighted by red arrows.

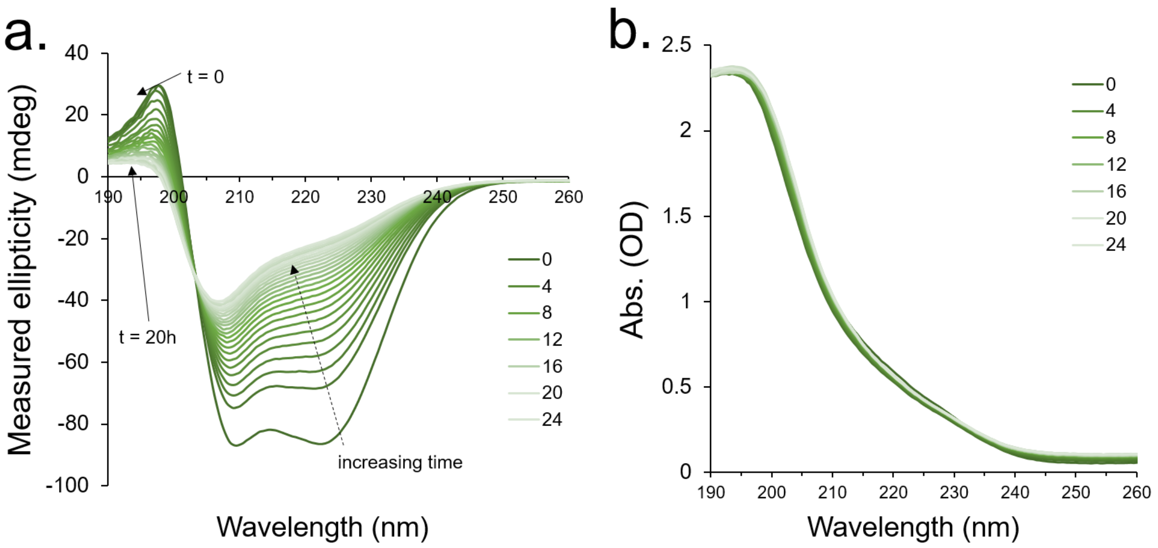

Figure S4. a) CD spectra of a a 5 wt. % HSA solution dissolved in 3M urea laminated between two quartz substartes over a 24h period and b) coresesponding UV absorbance profiles.

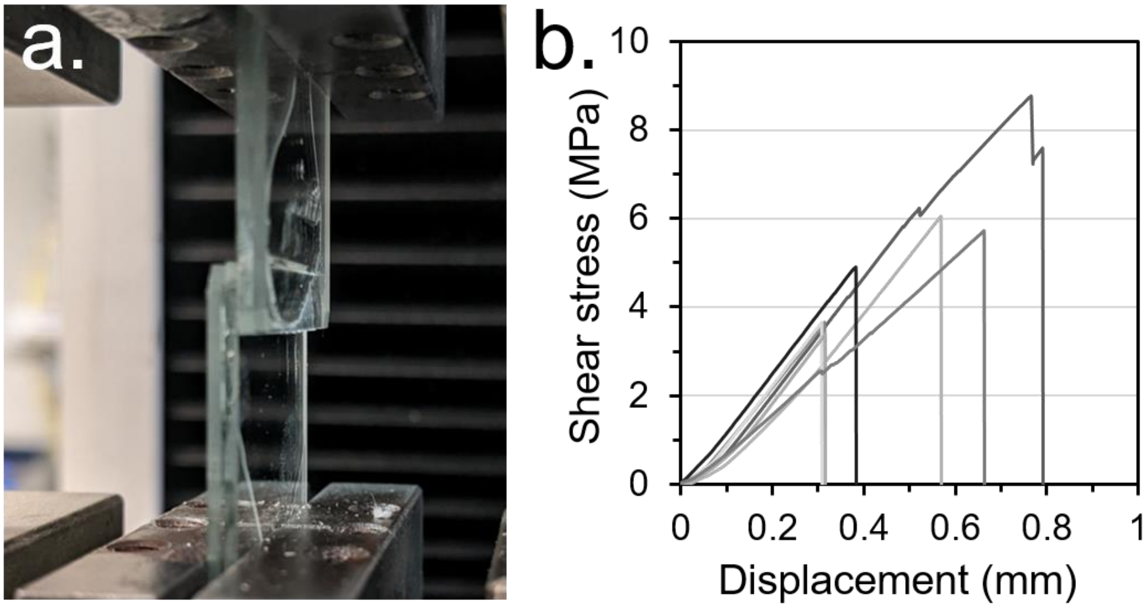

Figure S5. a) Visible light image of the set-up employed to test aqueous HSA as an adhesive on glass. b) Obtained stress vs. displacement curves.

Supplementary Table S1. Summary of advantages and disadvantages of various possible binder materials for Lunar or Martian regolith

Binder	Production	Advantages	Disadvantages
Human serum albumin (HSA)	*in vivo*	- No *in situ* synthesis equipment needed – astronauts will already be present<br>- 3D-printable<br>- Low temperature processing<br>- Non-toxic and non-flammable<br>- Required raw materials (food and water) already produced in surplus<br>- HSA has other uses (medical, emergency food)<br>- HSA-RBCs could be recycled for emergency consumption or use as soil for plant growth<br>- Water is released during curing (recoverable)<br>- Future missions could replace humans with animals to utilise same technology with fewer drawbacks (see below)<br>- HSA could also be produced recombinantly with appropriate technology (see below)	- HSA extraction could be psychologically and physiologically taxing on the crew<br>- Increased food consumption by crew needed<br>- Rate of HSA production limited by number of crew<br>- Extraction and purification equipment still needed<br>- HSA-RBCs could be subject to microbial degradation
Mammalian serum albumin (MSA)	*in vivo*	- 3D-printable<br>- Low temperature processing<br>- Non-toxic and non-flammable<br>- Required raw materials (biomass and water) already produced in surplus<br>- Water is released during curing (recoverable)<br>- Animals could consume waste cellulosic biomass and efficiently convert to manure for recycling<br>- Animals could provide a psychological boost to crew members<br>- Animals could produce other renewable resources (e.g. milk, eggs, wool)<br>- Animals could be consumed in an emergency	- Care of animals would be an additional workload for the crew<br>- Health and welfare of animals may be problematic<br>- Food, water and oxygen consumption by animals would compete with crew<br>- Animals could only perform menial tasks at best.<br>- Animals would make design of ship and Martian habitat more complex<br>- Animals or animal waste products in microgravity could be problematic<br>- MSA-RBCs could be subject to microbial degradation
Pulse albumins (PAs)	*in situ*	- No in situ synthesis equipment needed<br>- Possibly 3D-printable<br>- Low temperature processing<br>- Non-toxic and non-flammable<br>- Water is released during curing (recoverable)<br>- Required raw materials (pulses) could be produced as part of the food supply<br>- PA-RBCs could be recycled for emergency consumption or use as soil for plant growth	- Albumin production slower than serum albumin<br>- Protein extraction not as straight-forward as for serum albumin, requiring additional processing and purification equipment.<br>- Processing will require energy and produce waste, although waste could be recycled.<br>- Processing and purification equipment will add to mass (and cost) of mission, and could malfunction beyond repair<br>- PA-RBCs could be subject to microbial degradation<br>- Properties as a binder are unproven
Recombinant proteins (e.g. synthetic spider silk)	*in situ*	- 3D-printable (e.g. recombinant serum albumin)<br>- Bioreactor could have other uses (e.g. food, pharmaceuticals, or speciality chemicals)<br>- Proteins could be genetically engineered for superior properties<br>- Protein-RBCs could be recycled for emergency consumption or use as soil for plant growth<br>- Water is released during curing (recoverable)	- *In situ* synthesis equipment (bioreactor) would add significant mass, complexity and cost to a mission<br>- Higher water-, energy- and nutrient consumption to sustain microorganisms and bioproduction.<br>- Bioreactor could malfunction beyond repair<br>- Microbes may not perform well in a Martian environment (low gravity, high radiation)<br>- Protein-RBCs could be subject to microbial degradation<br>- Current technology requires substantial development before reliability, yield and waste generation within acceptable tolerances
Synthetic polymers	*ex situ*	- No in situ synthesis equipment needed<br>- Not restricted to Mars-applicable technology<br>- Advanced polymers could be employed using Earth-based technology	- Significant additional mass burden, adding to mission cost<br>- No possibility of making more on Mars
	in situ	- Depending on polymers that can be produced, potentially superior mechanical properties and durability than protein-based binders<br>- Potentially 3D-printable<br>- Large quantities could potentially be produced<br>- The synthetic polymers could have other uses<br>- Potentially recyclable	- *In situ* synthesis equipment would add significant mass (and cost) to mission<br>- Significantly higher water-, energy- and oxygen consumption – depending on polymer and technology.<br>- Equipment could malfunction beyond repair<br>- Technology requires significant development before Mars-ready<br>- Depending on polymer, may not be recyclable
Martian cement	*in situ*	- Large quantities could potentially be produced<br>- Significant know-how from Earth-based technology<br>- Good mechanical properties and durability<br>- Potentially 3D-printable	- Water consumed during curing (non-recoverable)<br>- Transport and processing equipment would add significant mass and may malfunction<br>- High temperature processing (400 – 1000 °C) will require significant energy<br>- Mineral deposits may be geographically sparse and require significant processing (milling, purification, calcination etc.)
Sintered or melted regolith	*in situ*	- Large quantities could potentially be produced from Martian regolith<br>- No water needed<br>- Good mechanical properties and durability	- Extremely high energy cost<br>- Additional energy generation equipment (e.g. solar panels, wind turbines) would be needed, adding to mass burden and costs.

			- Large bricks difficult to produce: adhesive/mortar needed to adhere smaller blocks
Sulfur	*in situ*	- Large quantities could potentially be produced from Martian regolith<br>- By-products from extraction could be utilised for other applications<br>- Curing does not consume water<br>- Mechanical properties could be improved with addition of terpenoids (essential oils) which could be grown or produced in a bioreactor	- Poorer mechanical properties limit applications<br>- Not easily 3D-printable<br>- Subject to sublimation under low pressures and elevated temperatures<br>- High temperature processing needed to extract sulfur from regolith<br>- Mining, milling, transportation and processing equipment will add to mass and complexity of mission – increasing costs<br>- Processing equipment may malfunction beyond repair
Frozen water	*in situ*	- Large quantities could potentially be produced if large water deposits can be exploited<br>- Water extraction technology will be employed for other applications (no additional technology or equipment would need to be developed)	- Poorer mechanical properties limit applications<br>- Not easily 3D-printable<br>- Extremely high water consumption will compete with other critical functions (food production, life support)<br>- additional water extraction equipment will add to mass (and cost) of mission<br>- Subject to sublimation under low pressures and elevated temperatures

Supplementary Table S2. Full breakdown of protein secondary over 20 h for 5 wt. % HSA laminated between quartz substrates based on CD measurements.

Time (h)	α-helix1 (%)	α-helix2 (%)	β-anti1 (%)	β-anti2 (%)	β-anti3 (%)	β-parallel (%)	β-turn (%)	Other (%)	NRMSD	Total α (%)	total β (%)
0	52.16	18.11	0	0	2.04	0	6.74	20.95	0.0151	70.27	8.78
1	47.54	17.99	0	0	2.59	1.02	8.55	22.3	0.0139	65.53	12.16
2	42.62	18.21	0	0	2.93	2.09	10.17	23.98	0.0135	60.83	15.19
3	37.17	17.49	0	0	3.37	3.78	12.05	26.13	0.0127	54.66	19.2
4	31.12	16.66	0	0	4	4.76	14.54	28.92	0.0122	47.78	23.3
5	22.85	14.17	0	0	4.18	7.61	15.89	35.29	0.0125	37.02	27.68
6	16.77	11.85	0	2.86	3.28	9.45	15.17	40.61	0.0133	28.62	30.76
7	13.26	9.81	0	5.17	2.59	11.02	14.21	43.94	0.0138	23.07	32.99
8	15.73	11.19	0	3.62	3.01	9.79	14.7	41.96	0.0131	26.92	31.12
9	14.27	10.76	0	4.5	3.01	10.41	14.36	42.68	0.0134	25.03	32.28
10	13.68	10.39	0	4.71	3.14	10.41	14.22	43.44	0.0139	24.07	32.48
11	13.1	9.74	0	4.53	3.39	10.65	14.14	44.45	0.0148	22.84	32.71
12	11.88	9.48	0	4.75	3.75	11.07	14.41	44.66	0.0162	21.36	33.98
13	11.44	9.07	0	4.8	3.85	11.06	14.35	45.43	0.0176	20.51	34.06
14	10.5	8.7	0	5.45	3.69	11.09	14.03	46.55	0.0179	19.2	34.26
15	9.2	8.13	0	6.16	3.57	10.26	14.06	48.61	0.0189	17.33	34.05
16	9.33	8.39	0	6.36	3.7	9.42	14.17	48.62	0.0195	17.72	33.65
17	8.84	8.13	0	7.41	4.77	8.41	14.4	48.03	0.0197	16.97	34.99
18	8.48	7.94	0	8.24	4.84	7.84	14.57	48.08	0.0207	16.42	35.49
19	7.83	7.76	0	8.82	5.29	7.19	14.75	48.35	0.0231	15.59	36.05
20	7.57	7.47	0	9.21	6.81	6.63	14.6	47.71	0.0224	15.04	37.25

Supplementary Table S3. Full breakdown of protein secondary for 0.1 wt. % HSA heated from 25 to 85 °C and back down to 25 °C.

Temp. (°C)	α-helix1 (%)	α-helix2 (%)	β-anti1 (%)	β-anti2 (%)	β-anti3 (%)	β-parallel (%)	β-turn (%)	Other (%)	NRMSD	Total α (%)	total β (%)
25	31.98	18.02	0.8	0.00	5.62	5.64	3.68	34.26	0.0331	50	15.74
35	29.8	16.1	1.64	0.00	10.03	9.89	2.48	30.06	0.0448	45.9	24.04
45	28.67	14.78	1.87	0.00	14.36	10.98	2.21	27.13	0.0564	43.45	29.42
55	27.99	15.11	1.83	0.00	16.05	8.54	3.13	27.35	0.0602	43.1	29.55
65	27.51	16.27	1.52	0.00	17.55	2.04	5.35	29.76	0.0602	43.78	26.46
75	23.37	14.3	0.58	0.00	18.96	0.97	8.93	32.89	0.0578	37.67	29.44
85	15.68	14.44	0	0.00	17.8	6.23	11.12	34.73	0.0544	30.12	35.15
75	16.57	14.5	0	0.00	18.65	6.56	10.31	33.41	0.054	31.07	35.52
65	17.28	14.7	0	0.00	18.78	6.64	9.71	32.9	0.0529	31.98	35.13
55	18.08	14.86	0	0.00	19.02	6.35	9.14	32.56	0.053	32.94	34.51
45	18.99	14.76	0.42	0.00	19.31	5.75	8.4	32.37	0.0563	33.75	33.88
35	19.52	14.75	0.68	0.00	19.42	5.64	8.14	31.85	0.0538	34.27	33.88
25	20.11	14.81	0.89	0.00	19.47	5.26	7.88	31.58	0.052	34.92	33.5

Supplementary References

1. B. G. Drake, S. J. Hoffman, and D. W. Beaty: in *IEEE Aerosp. Conf. Proc.* pp. 1–24.

2. S. N. Majhi and V. R. Nair: in *Microgravity Sci. Technol.* pp. 117–120.

3. Y. Reches: Concrete on Mars: Options, challenges, and solutions for binder-based construction on the Red Planet. *Cem. Concr. Compos.* **104**, 103349 (2019).

4. H. Roedel, I. R. Plata, M. Lepech, and D. Loftus: Sustainability assessment of protein-soil composite materials for limited resource environments. *J. Renew. Mater.* **3**(3), 183 (2015).

5. R. Raoufinia, A. Mota, N. Keyhanvar, F. Safari, S. Shamekhi, and J. Abdolalizadeh: Overview of albumin and its purification methods. *Adv. Pharm. Bull.* **6**(4), 495 (2016).

6. V. P. Grachev, A. Hoppe, D. I. Magrath, D. P. Thomas, D. Barrowcliffe, P. Brunko, N. Chariatte, J. Fischer, T. Golosova, H. J. Heiniger, A. G. Hildebrandt, F. Horaud, M. Koch, K. Komuro, M. Mozen, V. Ray, R. W. Reilly, M. Rodell, W. G. Van Aken, Q. H. Zhang, P. Corcoran, V. Grachev, C. Jersild, J. Koistinen, and D. Magrath: Requirements for the collection, processing and quality control of blood, blood components and plasma derivatives (Requirements for Biological Substances No. 27,

revised 1992). *World Heal. Organ. - Tech. Rep. Ser.* **840**(840), 34 (1994).

7. A. A. Menezes, J. Cumbers, J. A. Hogan, and A. P. Arkin: Towards synthetic biological approaches to resource utilization on space missions. *J. R. Soc. Interface* **12**(102), 20140715 (2015).

8. M. P. Bodiford, M. R. Fiske, W. McGregor, and R. D. Pope: in *A Collect. Tech. Pap. - 1st Sp. Explor. Conf. Contin. Voyag. Discov.* pp. 974–980.

9. H. Roedel, M. D. Lepech, and D. J. Loftus: *Earth Sp. 2014 Eng. Extrem. Environ. - Proc. 14th Bienn. Int. Conf. Eng. Sci. Constr. Oper. Challenging Environ.* 291 (2014).

10. S. L. Taylor, A. E. Jakus, K. D. Koube, A. J. Ibeh, N. R. Geisendorfer, R. N. Shah, and D. C. Dunand: Sintering of micro-trusses created by extrusion-3D-printing of lunar regolith inks. *Acta Astronaut.* **143**, 1 (2018).

11. S. Do, A. Owens, K. Ho, S. Schreiner, and O. De Weck: An independent assessment of the technical feasibility of the Mars One mission plan - Updated analysis. *Acta Astronaut.* **120**, 192 (2016).

12. A. Bujacz: Structures of bovine, equine and leporine serum albumin. *Acta Crystallogr. Sect. D Biol. Crystallogr.* **68**(10), 1278 (2012).

13. A. A. Menezes, M. G. Montague, J. Cumbers, J. A. Hogan, and A. P. Arkin: Grand challenges in space synthetic biology. *J. R. Soc. Interface* **12**(113), 20150803 (2015).

14. R. E. Wang, L. Tian, and Y. H. Chang: A homogeneous fluorescent sensor for human serum albumin. *J. Pharm. Biomed. Anal.* **63**, 165 (2012).

15. Z. Bahouth, B. Moskovitz, S. Halachmi, and O. Nativ: Bovine serum albumin-glutaraldehyde (BioGlue®) tissue adhesive versus standard renorrhaphy following renal mass enucleation: A retrospective comparison. *Ther. Adv. Urol.* **9**(3–4), 67 (2017).

16. Exolith Lab: LHS-1 Fact Sheet. (2021). Available at:

https://exolithsimulants.com/products/lhs-1-lunar-highlands-simulant.

17. Exolith Lab: MGS-1 Fact Sheet. (2021). Available at: https://exolithsimulants.com/products/mgs-1-mars-global-simulant.
